# Axonal Degeneration during Aging and Its Functional Role in Neurodegenerative Disorders

**DOI:** 10.3389/fnins.2017.00451

**Published:** 2017-09-04

**Authors:** Natalia Salvadores, Mario Sanhueza, Patricio Manque, Felipe A. Court

**Affiliations:** ^1^Center for Integrative Biology, Faculty of Sciences, Universidad Mayor Santiago, Chile; ^2^Fondap Geroscience Center for Brain Health and Metabolism Santiago, Chile

**Keywords:** axonal degeneration, aging, neurodegeneration, disease models, axonopathy

## Abstract

Aging constitutes the main risk factor for the development of neurodegenerative diseases. This represents a major health issue worldwide that is only expected to escalate due to the ever-increasing life expectancy of the population. Interestingly, axonal degeneration, which occurs at early stages of neurodegenerative disorders (ND) such as Alzheimer's disease, Amyotrophic lateral sclerosis, and Parkinson's disease, also takes place as a consequence of normal aging. Moreover, the alteration of several cellular processes such as proteostasis, response to cellular stress and mitochondrial homeostasis, which have been described to occur in the aging brain, can also contribute to axonal pathology. Compelling evidence indicate that the degeneration of axons precedes clinical symptoms in NDs and occurs before cell body loss, constituting an early event in the pathological process and providing a potential therapeutic target to treat neurodegeneration before neuronal cell death. Although, normal aging and the development of neurodegeneration are two processes that are closely linked, the molecular basis of the switch that triggers the transition from healthy aging to neurodegeneration remains unrevealed. In this review we discuss the potential role of axonal degeneration in this transition and provide a detailed overview of the literature and current advances in the molecular understanding of the cellular changes that occur during aging that promote axonal degeneration and then discuss this in the context of ND.

## Introduction

### Neurodegeneration during aging

The aging process is part of life and as such, it cannot be circumvented. However, much effort is currently devoted to understand the molecular changes that occur during aging and cause pathologies, in an attempt of being able to modify them to have the possibility of living a healthier aging.

The effects of aging on the brain are multiple and importantly, age constitutes the main risk factor for the development of neurodegenerative disorders (NDs) such as Alzheimer's disease (AD), Parkinson's disease (PD), and amyotrophic lateral sclerosis (ALS), which are characterized by progressive neuronal death and loss of specific neuronal populations. Considering the constant increase in life expectancy, NDs are nowadays an important problem for the society and our efforts to understand the mechanisms underlying these disorders has not been sufficient to provide a definitive help to the millions of patients worldwide.

For a better comprehension of the molecular and cellular changes that occur during aging, seven pillars of aging were defined, which are common processes involved in most chronic disorders that take place in an aging organism. These seven pillars are proteostasis, adaptation to stress, inflammation, stem cells and regeneration, epigenetics, metabolism, and macromolecular damage (Kennedy et al., 2014). Notably, changes in these cellular events are common to most NDs, suggesting that similar mechanisms might at least partially explain different age-related diseases. Even though NDs share phenotypic commonalities such as protein aggregation, cellular stress responses, and failure in RNA metabolism, it is still not clear why heterogeneous responses to similar genetic and environmental stimuli take place in different neuronal populations. Understanding the molecular basis of these pillars of aging and the timeframe in which they are activated could help us to tackle pre-symptomatically NDs and avoid irreversible cellular changes.

Axonal degeneration, which occurs at early stages of NDs, also takes place as a consequence of normal aging. Indeed, many cellular processes that are altered with advanced age have shown to contribute to axonal pathology. Importantly, the degeneration of axons represents an early event during the development of NDs, preceding both cell death and the onset of clinical symptoms, which has important therapeutic implications. Although, the molecular basis of the transition that makes an individual to develop neurodegeneration with advanced age is currently unknown, increasing evidence support the potential role of axonal degeneration in this transition, which is the focus of this review. An outline of the mechanisms associated to axonal degeneration is presented below, followed by a detailed overview of the literature and current advances in the molecular understanding of the cellular changes that occur during aging and its relationship with axonal degeneration.

### Axonal degeneration overview

The process of axonal degeneration is an essential developmental event that consists in the selective destruction of axons (Schuldiner and Yaron, 2014). Moreover, axonal degeneration also occurs as a consequence of aging and represents a feature of NDs, constituting an important contributor to neuronal dysfunction (Neukomm and Freeman, 2014). Notably, the evidence indicates that axonal degeneration is an early event in NDs, taking place previous to neuronal cell death (Deckwerth and Johnson, 1994; Adalbert and Coleman, 2013).

Axonal degeneration is an evolutionary conserved process that can be activated by different stimuli including mechanical damage, axonal transport defects or by drugs used for chemotherapy. Although, the exact molecular and cellular pathways by which axonal degeneration occurs remain to be fully clarified, key contributing factors have been identified in the last decade and crucial findings have contributed to elucidate the mechanisms involved. Important clues have been obtained by studying Wallerian degeneration (WD), which correspond to degeneration of isolated axon after their mechanical transection. Furthermore, studies in the mouse strain Wld^S^, which presents delayed axonal degeneration after injury, has been crucial to understand the mechanisms associated to axonal degeneration, and its functional relevance in NDs (Mack et al., 2001). After nerve transection, desomatized wild type axons undergo three phases: a latent phase, axonal fragmentation and axonal disintegration. The latent phase stills poorly understood but it is known that axons remain apparently normal for 1–2 days in mice after nerve injury (Court and Coleman, 2012), and can still conduct action potential (Moldovan et al., 2009). In the last stage, all the structures inside the axon are degraded. Disintegration of axonal cytoskeleton is followed by myelin degradation and macrophage infiltration that clear cell debris (Coleman, 2005).

Genetic analysis of the Wld^S^ mice unveiled that this natural mutation corresponds to a neomorphic one, that overexpresses a chimeric protein formed by fusion of the N-terminus of the E4 ubiquitin ligase Ube4b with the complete sequence of nicotinamide mono nucleotide adenylyltransferase 1 (Nmnat1; Coleman and Freeman, 2010). Axonal protection observed by up-regulating Nmnat1 is linked to mitochondrial metabolism (Avery et al., 2009; Fang et al., 2012), and the main mechanism does not seem to be the enzymatic production of NAD by Nmnat1 but most likely the action of downstream targets of this protein (Sorci et al., 2007; Coleman and Freeman, 2010). Recently, a loss-of-function mutation in the Sterile alpha and Toll/interleukin receptor (TIR) motif-containing protein 1 (Sarm1) was found, which cell-autonomously suppresses WD, confirming that this process is indeed an active program (Osterloh et al., 2012). SARM1 is a conserved mediator of WD, acting through the dimerization of the TIR domain to rapidly deplete NAD^+^ in injured axons to trigger degeneration (Gerdts et al., 2015; Summers et al., 2016; Essuman et al., 2017). This mechanism explains NMNAT1 suppression of WD as this protein blocks the injury-induced NAD^+^ consumption caused by SARM1, a mechanism that seems to be more important than the altered NAD^+^ production caused by NMNAT1 (Sasaki et al., 2016). These findings open the possibility to identify novel molecules actively involved in the process that could lead ultimately to a deeper characterization and novel therapeutic targets for neurodegeneration.

We have demonstrated that mitochondrial dysfunction is a key process associated to axonal degeneration (Barrientos et al., 2011). The degeneration of axons was shown to be associated to the formation of the mitochondrial permeability transition pore (mPTP) between the inner and outer mitochondrial membrane. mPTP formation triggers the mitochondrial permeability transition (mPT), which leads to an increase in axonal reactive oxygen species (ROS) followed by intra-axonal calcium release (Calixto et al., 2012; Villegas et al., 2014). Interestingly, blocking mPTP either pharmacologically or genetically, by removal of the mPTP component Cyclophilin D (CypD), significantly delays axonal degeneration (Barrientos et al., 2011). Notably, formation of the mPTP has been linked to the pathogenesis of NDs including AD (Du et al., 2008), PD (Martin et al., 2014), and ALS (Martin et al., 2009) and has been suggested as a potential therapeutic target for these diseases.

Increasing evidence suggest that axonal degeneration occurs before cell body loss and notably, previous to the onset of clinical symptoms in different models of age-related diseases including ALS (Dadon-Nachum et al., 2010), PD (Tagliaferro and Burke, 2016), and AD (Adalbert and Coleman, 2013). Hence, the understanding of the molecular and cellular mechanisms underlying this potentially reversible phase is critical for the development of therapeutic strategies aimed at the prevention and intervention of these disorders. Multiple molecular and cellular changes that occur during the process of aging can contribute to the accumulation of axonal damage, which is a prominent histopathological feature of the aging brain. Importantly, these cellular changes are common to almost all NDs, suggesting that similar mechanisms participate in the onset and development of these disorders. In the following section, we discuss how each of these changes contribute to the alteration of axonal integrity.

## Molecular mechanisms involved in aging and their relationship with axonal degeneration

**Figure 1:**
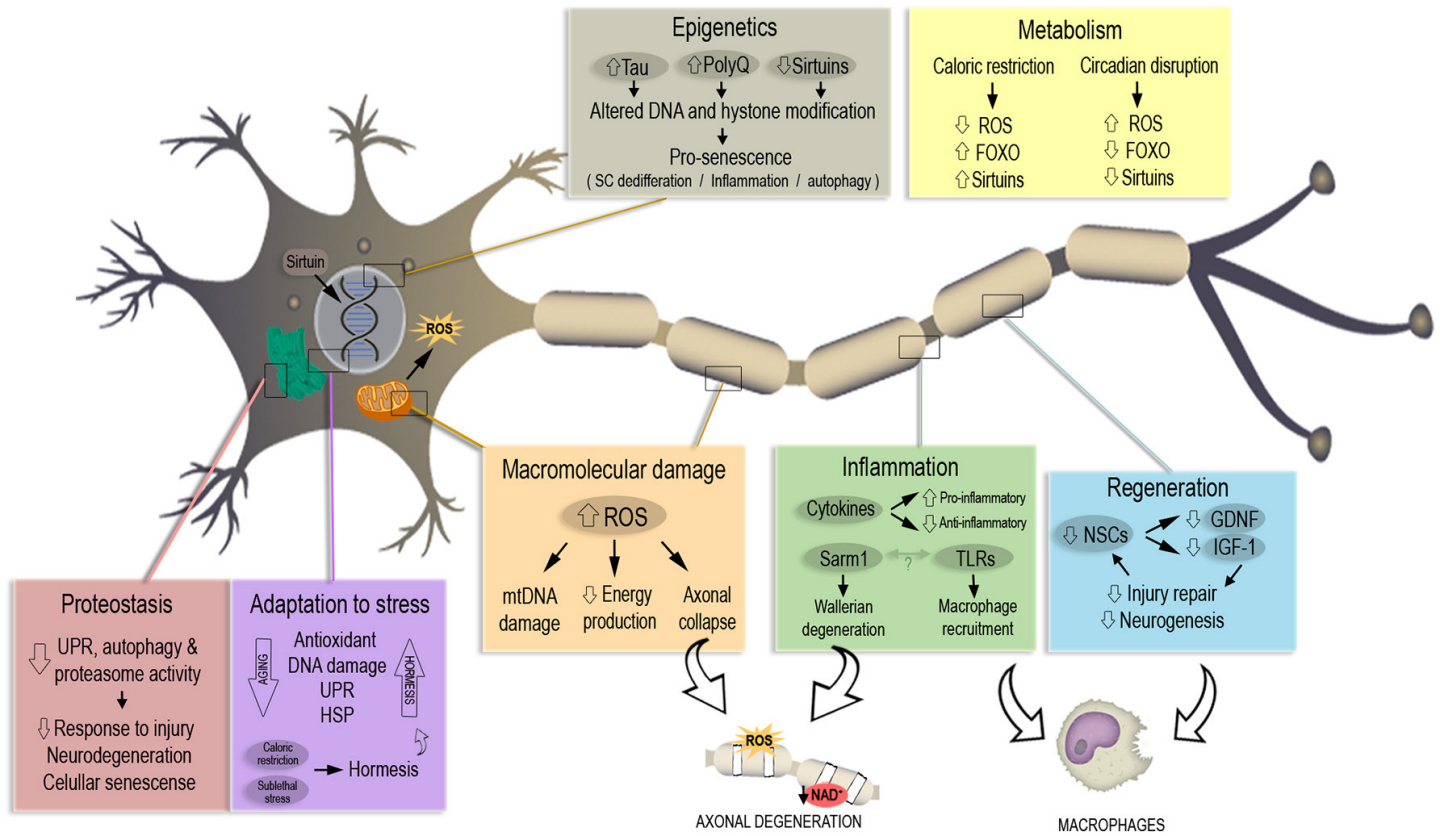
The seven pillars of aging in the context of neuronal and axonal degeneration. Each pillar associated to the aging process is represented in a colored box. Most relevant pathways and molecules misregulated during aging are highlighted in each box, altogether with the consequences in neuronal senescence and axonal degeneration. A misregulated response to macromolecular damage and inflammation lead to increased ROS and a decrease in available NAD^+^, triggering axonal degeneration. Aging also decreases the number of neuronal stem cells (NSCs) and their regenerative capability. Caloric restriction works in a protective way against aging with a mechanism opposite to the one observed with the age-linked disruption of circadian rhythm. Altered DNA modification and repair trigger pro-senescence phenotypes that lead to neuronal death, same phenotype induced by decrease of response to stress stimuli and oxidative damage.

The urgency to extend healthspan, the period of healthy life preceding the development of age-related chronic diseases, has been recently highlighted. From this perspective, the field of Geroscience has invested increasing efforts to understand the mechanisms that underlie lifespan alteration, linking aging, and chronic diseases with the final aim of developing therapies for age-associated diseases (Kennedy et al., 2014). In this section we will review latest findings on each of the seven pillars of aging (Figure 1) with the aim of understanding whether they are related to axonal degeneration and how this event can be shifting healthy aging toward pathological aging with prevalence of chronic and neurodegenerative diseases.

### Adaptation to stress

In response to harmful stimuli, protective mechanisms that trigger adaptive responses can be activated to maintain cell homeostasis. If such noxious stimuli persist, programmed cell death is usually activated to eliminate damaged cells (Fulda et al., 2010). Whether cells react by triggering protective or destructive pathways depends on different factors including the nature and extent of the stress. Cells can respond to harmful stimuli in a number of ways and these adaptive responses include, among others, antioxidant defense mechanisms, the unfolded protein response (UPR), the heat-shock response and the DNA damage response (Fulda et al., 2010). As an example, it has been shown that following hypoxic injury, activation of the hypoxia inducible factor 1 (HIF-1) induces the expression of several genes that can promote cell survival and tissue adaptation by increasing blood supply and oxygen delivery to the injured tissue (Majmundar et al., 2010).

Compelling evidence indicate that the capability to induce an effective response following environmental and cellular injury decreases with the progression of aging. Hence, as organisms age, along with a general deterioration of cellular function, there is a decline in the stress responses that promote homeostatic repair. Examples of this diminished capability to compensate the altered cellular homeostasis include diminished HIF-1 activity (Majmundar et al., 2010), decreased heat shock response (Fargnoli et al., 1990) and altered response against DNA damage (Druzhyna et al., 2008). Notably, the concept of hormesis has emerged in the context of homeostasis, associated to a phenomenon in which an organism that has been subjected to sub-lethal stress such as caloric restriction, can engage signaling pathways that increase the neuronal capabilities of stress resistance against oxidative stress, mitochondrial disruption, protein misfolding, and DNA damage, thus reducing physiological decline and leading to life span extension (Mattson, 2008). By instance, studies performed in different models including mice (Miller et al., 2017), monkeys (Mattison et al., 2017), and humans (Prehn et al., 2016) have demonstrated that dietary energy restriction can protect against brain degeneration, promote cognitive improvement and increase life span (discussed in more detail in Section Metabolism).

Importantly, studies aimed at determine the effect of caloric restriction in NDs have shown positive outcomes using models of AD (Wu et al., 2008), PD (Maswood et al., 2004), and Huntington disease (HD; Duan et al., 2003). Several mechanisms have been demonstrated to contribute to this protective stress response and includes the Ca^2+^–cyclic AMP response element-binding protein (CREB) pathway, the sirtuin–forkhead box O (FOXO) pathway and the nuclear regulatory factor 2 (NRF2)–antioxidant response element (ARE) pathway (Mattson, 2008; Stranahan and Mattson, 2012). Similarly, the experimental activation of other adaptive pathways, with the purpose of testing their potential therapeutic benefit for different NDs, has been recently investigated. In this line, the role of UPR activation, which is a main player of the proteostasis network, in the development of NDs such as AD (Duran-Aniotz et al., 2017) and PD (Valdes et al., 2014) has revealed interesting results. Recent evidence has shown an important link between the UPR and axonal degeneration and will be discussed in detail in the following section. Additionally, autophagy constitutes an essential component of the adaptive response to cell stress that helps to maintain cellular homeostasis and quality control. This mechanism is essential to regulate the axonal proteome and maintain axonal homeostasis by eliminating damaged organelles and protein aggregates (Komatsu et al., 2007; Maday and Holzbaur, 2014). However, the experimental induction of autophagy can result in protective or harmful effects to axons, which appears to depend on the context of the experimental setting. Indeed, there are reports demonstrating detrimental effects of autophagy activation on axonal integrity (Park et al., 2008; Kim et al., 2009; Liu et al., 2010; Cheng et al., 2011; Wakatsuki et al., 2017), while other studies have shown a protective effect (Komatsu et al., 2007; Launay et al., 2014; He et al., 2016). Despite the opposed outcomes, these studies strongly suggest that autophagy represent an important process involved in axonal maintenance and degeneration.

### Proteostasis

The maintenance of protein homeostasis (referred to as proteostasis), which involve the correct synthesis, folding, trafficking, secretion, and degradation of proteins, relies on a network of different mechanisms and pathways that include the UPR, the heat-shock response, the autophagy pathway, the ubiquitin–proteasome system, chaperones and the endoplasmic reticulum (ER)-associated degradation machinery (ERAD). This cellular machinery maintains the equilibrium of the proteome and prevents the accumulation of misfolded proteins (Labbadia and Morimoto, 2015).

Compelling evidence has demonstrated a decline in the homeostatic capacity of the proteostasis network with increasing age. By instance, several studies have demonstrated that the levels of chaperones are markedly decreased with age in different organs including the brain (Paz Gavilán et al., 2006; Hussain and Ramaiah, 2007; Naidoo et al., 2008; Walther et al., 2015). Chaperoning activity can also be influenced by age-related changes such as energy failure due to mitochondrial damage, which affect the function of ATP-dependent chaperones (Brehme et al., 2014). Additionally, a number of studies have demonstrated aging-associated defects on autophagy (Cuervo and Dice, 2000; Lipinski et al., 2010; Rubinsztein et al., 2011) and proteasome activity (Bulteau et al., 2002; Ferrington, 2005; Dasuri et al., 2009; Keller et al., 2015), which contribute to the decline of brain proteostasis. Notably, there is evidence indicating that either manipulating the proteostasis network or preventing its deterioration, can induce a slowdown in the aging progression in different animal models (Ruan et al., 2002; Henis-Korenblit et al., 2010; Kruegel et al., 2011; Vilchez et al., 2012; Owusu-Ansah et al., 2013; Labunskyy et al., 2014). As an example, overexpression of the mitochondrial heat shock protein 22 leads to increased lifespan in *Drosophila* (Morrow, 2004). Another study showed that activation of autophagy by overexpression of Atg5 was able to extend the lifespan of mice (Pyo et al., 2013). Similarly, enhancement and activation of the 20S proteasome in *Caenorhabditis elegans*, resulted in life span extension (Chondrogianni et al., 2015).

A common feature of aging and NDs is the misfolding and accumulation of protein aggregates in the brain, a slow process that initiates decades before clinical symptoms manifest. The exact cause that leads to protein aggregation in the brain is unknown, although deregulation of protein homeostasis through perturbation of the ER has been shown to be involved (Martínez et al., 2017). The build-up of misfolded proteins at the ER, a condition known as ER stress, triggers the UPR signaling pathway in order to restore proteostasis and promote cell survival (Sidrauski and Walter, 1997; Harding et al., 1999; Haze et al., 1999). Disruption of this adaptive pathway has been associated with advanced age and it has emerged as a key contributor to the pathogenesis of NDs such as AD (Cissé et al., 2010, 2016; Li et al., 2013), prion disease (Moreno et al., 2012, 2013), ALS (Hetz et al., 2009), PD (Imai et al., 2001; Cooper et al., 2006; Valdes et al., 2014), and HD (Vidal et al., 2012; Zuleta et al., 2012).

Recent evidence indicates that activation of ER stress plays a critical role in the neuronal response to axonal injury in both peripheral and CNSs (Smith and Mallucci, 2016). Indeed, numerous studies have shown activation of the UPR upon axonal damage in different type of cells including Schwann cells (Mantuano et al., 2011), motoneurons (Penas et al., 2011), retinal ganglion cells (Hu et al., 2012), and in sensory neurons of the dorsal root ganglia (Ying et al., 2015). Interestingly, pharmacological and genetic manipulation of components of the UPR pathway has shown to induce cognitive improvement and motor recovery in disease and nervous system injury models, respectively (Li et al., 2013; Valenzuela et al., 2016). By instance, studies from our group have demonstrated a protective role of the transcription factor X-Box-binding protein 1 (XBP1), a major regulator of the UPR, following nervous system damage. After spinal cord injury, XBP1-deficient mice presented significant impairment of locomotor recovery when compared with control mice. Notably, local administration of active XBP1 by gene therapy to the injured area enhanced locomotor recovery (Valenzuela et al., 2012). Looking at the functional role of the UPR in locomotor recovery following peripheral nerve injury, our group demonstrated that genetic ablation of Xbp1 induced a delay in locomotor recovery after injury. Conversely, overexpression of XBP1s in transgenic mice and local XBP1s gene transfer to neurons of wild-type mice, increased axonal regeneration and locomotor recovery (Oñate et al., 2016). Additionally, the protective effects of XBP1 activation on neuronal death, following axonal injury, were shown in a model of optic nerve crush. Axotomy triggered transient activation of the inositol-requiring enzyme 1α (IRE1α)/XBP1 pathway, and overexpression of the transcription factor strongly protected neurons from apoptosis (Hu et al., 2012). In a more recent study, using mouse models of traumatic optic nerve injury and glaucoma, the same group demonstrated that inhibition of eukaryotic translation initiation factor 2α-C/EBP homologous protein and XBP1 activation synergistically protect retinal ganglion cell axons and preserve visual function (Yang et al., 2016). Together, this evidence highlights the important role of the UPR signaling pathway in the processes of axonal degeneration and regeneration.

### Inflammation

Even though activation of immune responses and inflammatory processes are consistently linked to aging (Chung H. Y. et al., 2009), it has been difficult to define the role of inflammation in neurodegeneration (Ransohoff, 2016). Inflammation corresponds to the protective response by which immune cells react in a balanced way against unexpected cells or debris (Karin and Clevers, 2016). For example, inflammation constitutes a primary response against pathogenic microorganisms in the intestinal epithelium. Metabolic changes observed in aging cells (DNA damage, loss of proteostasis, stress signals) affect the immune response against pathogens in the intestine and it is likely the cause why elderly individuals are more susceptible to infectious diseases and changes in health and lifespan (Ayyaz and Jasper, 2013). Another metabolic change that occurs in aging cells is the redox imbalance, which can be caused by weakness of the anti-oxidative defense system that cannot cope with the increased production of reactive species (Chung H. Y. et al., 2009). Notably, the increased lifespan observed with the anti-oxidant action of caloric restriction is associated to the modulation of pro-inflammatory signals such as NF-kB, TNF-α, and interleukins (Kim et al., 2002; Zou et al., 2004). Importantly, aging is associated with abnormal inflammatory responses in the brain, where the levels of pro-inflammatory cytokines are elevated, and the anti-inflammatory ones are reduced (Ye and Johnson, 1999; Sierra et al., 2007; Cribbs et al., 2012).

The nervous system is able to generate immune responses, and most of these processes are commanded by microglia and dendritic cells (Carson et al., 2006). Macrophages dominate sites of CNS injury where they promote both injury and repair. These cells are classified into the proinflammatory, neurotoxic M1 cells, and the M2 cells, which promote axon growth and remyelination (Kigerl et al., 2009). Morphologically, inflammation in the CNS is characterized by a different shaped microglia, which looks hypertrophic after an acute damage, or dystrophic after aging and neurodegeneration (Ransohoff, 2016). On the other hand, following injury to the nervous system, an important event is the inflammatory response associated to WD. In this process, glial cells fragment axons, isolate and convert myelin into lipid droplets even before the arrival of macrophages (Stoll et al., 1989). Then, the levels of cytokines and chemokines such as TNF-α and IL-1 are upregulated, leading to macrophage recruitment (Gillen et al., 1998; Liefner et al., 2000).

Multiple sclerosis (MS) is perhaps the most classic neurodegenerative disease associated to inflammation. MS is an autoimmune disease triggered by CD4^+^ T helper lymphocytes and characterized by demyelination scattered throughout the CNS (Lucchinetti and Bruck, 2004). Mitochondria plays an important role in the pathomechanism of MS, affecting the normal relation between axons and glia through several defects including Ca^2+^ imbalance caused by excessive proinflammatory cytokines, deregulation of oxidative stress, impaired energy production and mitochondrial autophagy (Patergnani et al., 2017). These processes are in part mediated by the formation of the mPTP, which interestingly, has been strongly linked to axonal degeneration (Barrientos et al., 2011; Villegas et al., 2014). In fact, blocking mPTP formation has been proposed as a therapy for MS, with different compounds being currently tested in clinical trials (Su et al., 2012; Shirani et al., 2016).

Another interesting connection between inflammation, aging and axonal degeneration was made after the discovery that SARM1 is required for the degeneration of axons (Osterloh et al., 2012). Sarm1 is a pro-degenerative molecule that works after injury to trigger degeneration through a loss of NAD^+^, which is mediated by its TIR domain (Gerdts et al., 2015; Summers et al., 2016; Essuman et al., 2017). A crucial aspect of the mechanism is that Sarm1 cell-autonomously triggers axonal degeneration, a surprising finding considering the role of this molecule in immune responses (Carty et al., 2006). Sarm1, Myd88, and Trif are adaptor molecules for Toll-like receptors, and as they are expressed in neurons, they are able to produce cytokines in response to pathogen infection (Chen et al., 2011; Lin et al., 2014). Interestingly, Sarm1 is not expressed in glial cells (Lin et al., 2014) and it is evolutionarily distinct from other proteins carrying TIR domains (Malapati et al., 2017), suggesting that its complete role is not yet fully understood. Sarm1 colocalizes with mitochondria (Panneerselvam et al., 2012) and regulates cell death after glucose and oxygen deprivation, recruiting JNK3 to the mitochondria (Kim et al., 2007; Mukherjee et al., 2013). Also, this protein was found in a genome-wide screening as an activator of PMK-1, a *C. elegans* p38-related kinase involved in stress-induced detoxification, oxidative stress and aging (Crook-McMahon et al., 2014). Notably, over a decade ago Sarm1 was proposed as a candidate gene implicated in the onset of hereditary inflammatory diseases, after analyzing family-based human linkage disequilibrium studies (Mink and Csiszar, 2005). Recently, mice lacking the Sarm1 protein showed resistance to distal axonal degeneration in a model of chemotherapy induced peripheral neuropathy (Turkiew et al., 2017). These studies, altogether with the recent characterization of the TIR domain, open novel opportunities to use Sarm1 as a target for therapeutic approaches for neuropathies.

### Stem cells and regeneration

Regeneration of tissues after injury requires in most cases, the presence of functional stem cells, the population of cells able to self-renew and the primal source of differentiated cell types. As all cells, Stem cells can be target of damaging mechanisms that can affect their function, decrease their viability and ultimately, compromise their ability to produce new cell lines. The damage received by stem cells is the basis of one of the most accepted theories of aging, which suggests that aging at the organism level is caused by exhaustion of stem-cell populations and the loss of regenerative responses after damage (Ruzankina et al., 2007). Several age-associated processes have been linked to the affected function of stem cells, including telomere shortening (Ferron et al., 2009), cellular senescence (Molofsky et al., 2006), and other interconnected pillars of aging such as epigenetic (Sun et al., 2014), metabolism (Deng et al., 2015), and proteostasis (Fredriksson et al., 2012).

In the nervous system, neural stem cells (NSCs) are responsible for neurogenesis and neuron replenishment within limited areas of the CNS. Many approaches have been tested to use NSCs injection in specific regions of the brain as a therapeutic intervention for NDs. By instance NSCs injected into the subiculum or hippocampus of two different transgenic AD mouse models decreased Aβ pathology and improved synaptic deficits (Blurton-Jones et al., 2014). Similar results were obtained in the P301S-tau model, where injection of NSCs that differentiated into astrocytes, increased glial-derived neurotrophic factor (GDNF) production and led to neuroprotection (Hampton et al., 2010). In contrast, it has been more difficult to observe an improvement in PD after injection of NSCs in the substantia nigra (Lindvall, 2013), and in HD after injection of NSCs in the striatum (Cicchetti et al., 2009). However, just as in AD, the increased production of neurotrophins such as IGF-1 and GDNF could be key to reach an improvement in motor and cognitive response in both diseases. Therefore, transplantation of NSCs that stimulate neurotrophin production appears to be a promising therapeutic intervention for NDs (Marsh and Blurton-Jones, 2017).

NSCs have been also used to improve axonal regeneration in the peripheral nervous system (PNS) after injury or nerve transection. WD in axons is a required initial step for regeneration (Martin et al., 2010) and after axonal damage, Schwann cells switch from a myelinating to a phagocytic phenotype and recruit macrophages to initiate the regenerative process (Fairbairn et al., 2015). Following sciatic nerve injury in rats, injection of NSCs transfected with two recombinant vectors containing either brain-derived neurotrophic factor (BDNF) gene or GDNF gene increased myelination and induced functional recovery (Fu et al., 2011). Similarly, a silicon conduit filled with NSCs and NGF connecting a sciatic nerve injury was used to increase axon myelination and induce functional recovery in rats (Xu et al., 2012). It will be interesting to follow the next clinical trials using stem cells and specially, how they manage the current difficulties in the translation to patients, including heterogeneity of lines and techniques (Marsh and Blurton-Jones, 2017).

### Epigenetics

The fact that individuals with similar genetic backgrounds can age very differently constitutes an intriguing situation. Epigenetics involve the understanding of the mechanisms that allow individual cells to translate their genome differentially under functional and stable conditions in a multicellular organism (Schwartzman and Tanay, 2015). These mechanisms include DNA methylation, histone modification and chromatin accessibility, and they provide a different level of control for the genetic expression in each cell of the organism harboring the same genetic information at the DNA level. DNA methylation occurs at the 5′ position of a cytosine, preferentially when it is followed by a guanine (CpG context) and it is a major and dynamic mechanism for differential gene expression between tissues and cell-type differentiation (Boyd-Kirkup et al., 2013). On the other hand, histone modification through methylation, phosphorylation, acetylation and ubiquitylation, is another dynamic way to control gene expression, regulating the balance between the accessible euchromatin and the compacted heterochromatin, and with this, the facilitated transcription of specific DNA regions.

Both DNA methylation and histone modification have been linked to aging, supporting one of the original theories of aging: the increased difficulty of cells to express genes with aging due to changes in the DNA, especially the ones related to more relevant pathways for aged cells such as autophagy (Madeo et al., 2015). The modification of histones is a process affected by age. For instance, the protective effect of Sirtuins and Polycomb proteins through deacetylation and methylation respectively, is lost in old cells triggering the upregulation of NFkB and pro-senescence genes like p16 (Rando and Chang, 2012). Decreased levels of methylation were found in inflammatory genes such as TNF and iNOS when DNA from old blood cells was sequenced (Gowers et al., 2011). Also, hypermethylation was found in CpG islands of promoter regions of DNA from aged cells (Christensen et al., 2009), in DNA-binding factor genes in human brain (Hernandez et al., 2011) and in genes associated to development and differentiation such as FGF17, FZD1, and FZD7 (Salpea et al., 2012).

Epigenetic events in the context of neurodegeneration and axonal degeneration are less described than in aging, in part because studying epigenetic changes in the brain is particularly difficult. Cell heterogeneity and different functional states of neuronal populations makes epigenetics studies in the brain harder than other tissues (Maze et al., 2014). The microtubule-associated protein tau, a main player in AD pathomechanism, triggers heterochromatin relaxation in transgenic flies and AD patients in a mechanism mediated by oxidative stress and DNA damage (Frost et al., 2014). DNA remodeling increases the transcription of genes normally silent as compared to control transcriptional profiles, suggesting that this epigenetic effect may work as a potential therapeutic target for AD. Similar clues have been found in the study of HD, another neurodegenerative disease caused by the expansion of CAG repeats coding for glutamine (PolyQ) in the huntingtin gene. PolyQ expansions in this gene have been linked to DNA remodeling and histone modifications (Steffan et al., 2001; Sadri-Vakili et al., 2007), changes in DNA methylation and transcription of key neuronal-specific genes (Ng et al., 2013; Wood, 2013), and alteration of ncRNAs (Johnson et al., 2008; Lee et al., 2011). Expansions in C9orf72, the gene that is most commonly linked to ALS, include CpG islands that are hypermethylated in tissue from ALS patients (Xi et al., 2015), and altered histone methylation pattern causes reduction on the C9orf72 mRNA expression in the patient's brain (Belzil et al., 2014).

As explained above, WD after axonal injury is associated to dedifferentiation and proliferation of Schwann cells. Proliferation is regulated through an epigenetic effect of the histone demethylase Jmjd3, which after injury activates the Ink4a/Arf locus to switch off proliferation and trigger the senescence program (Gomez-Sanchez et al., 2013). The high plasticity observed in Schwann cells could also play an important role in the events leading to neurodegeneration. Current research efforts are focusing on trying to properly define the link between disease-linked mutations and the temporality of epigenetic effects. Hopefully, that connection could be used as a potential biomarker to determine pre-clinically the progression of neurodegenerative disorders.

### Metabolism

In this section, we will focus on two topics that, even though are connected to previously described mechanisms, can still provide novel views to understand the connection between aging and degenerative mechanisms in the neuron. These topics are nutrigenomics or the effect of the food and nutrients on gene expression (Grayson, 2010), and the regulation of circadian clocks and sleep patterns (Musiek and Holtzman, 2016).

Nowadays, the relevance of specific and personalized diets, with the aim of improving the quality and extension of life, is getting common. However, the beneficial outcome of specific types of food or a reduction on caloric intake, not only applies to lifespan extension (Madeo et al., 2015) but also has been associated to axonal degeneration (Speakman and Mitchell, 2011). Caloric restriction extends lifespan through different mechanisms that include increased autophagy and activation of mTOR and FOXO (Galluzzi et al., 2014), reduction of mitochondrial ROS production (Ash and Merry, 2011), mitochondrial biogenesis via upregulation of PGC-1a (Nisoli et al., 2005) and activity of Sirtuins (Jasper, 2013). Sirtuins are deacetylases that catalyze the consumption of NAD^+^, and are required for lifespan extension after supplementing nicotinic acid (a source of NAD^+^). Sirtuins have been extensively studied with contrasting results. In flies and worms, Sirt1 was linked with extension of lifespan (Rogina and Helfand, 2004; Viswanathan et al., 2005), a discovery that was later challenged (Burnett et al., 2011).

Not that well-studied, at least in a direct way, is the effect of caloric restriction on axonal degeneration. Previous studies performed by our group using a genetic *mec-4d C. elegans* model of axonal degeneration and a mouse model of acute injury, demonstrated that caloric restricted diet and systemic antioxidant treatment protected both models from axonal degeneration, which was associated with decreased oxidative damage. Moreover, downregulation of the Insulin/IGF-1-like signaling (IIS) pathway protected neurodegeneration in a DAF-16/FOXO–dependent manner (Calixto et al., 2012). As mentioned above, FOXO is an important player in lifespan extension acting as an effector of the stress-response JNK pathway, both antagonizing IIS and working together with the TOR pathway as a molecular switch between growth promotion and lifespan extension according to nutrient availability (Wang et al., 2005). Importantly, a phase 2 trial based on dietary restriction proved to be successful against metabolic syndrome through reducing glucose and circulating IGF-1 (Wei et al., 2017). Using another variation of dietary restriction (intermittent fasting), improvements in motor performance were observed in a mouse model of neuropathic pain (Madorsky et al., 2009) and in a spinal cord injury mouse model (Jeong et al., 2011), further supporting the effect of caloric restriction in neuronal health. Notably, SIRT2 was also linked to axonal degeneration (Araki et al., 2004) and WD (Suzuki and Koike, 2007) in a mechanism that involves tubulin deacetylation and a delay on axonal degeneration. However, the potential role of SIRT2 and NAD+/NADH balance in WD was discarded in *Drosophila*, as downregulation of this enzyme did not induce spontaneous degeneration and did not suppress the ability of Wld^S^ to slow axonal degeneration *in vivo* (Avery et al., 2009).

In addition, emerging evidence indicate that a ketogenic diet, which consists of high fat, adequate protein and low carbohydrate intake, can improve motor and cognitive performance in NDs. By instance, administration of ketogenic diet to transgenic ALS mice resulted in higher motor neuron survival and an in motor function improvement when compared to control mice (Zhao et al., 2006). Another study performed by the same group in transgenic ALS mice fed with caprylic triglyceride showed protection from spinal cord motor neuron loss and improved motor performance (Zhao et al., 2012). Similar positive results have been obtained in AD models. For example, the toxic effects of Aβ on hippocampal neurons were prevented by addition of β-hydroxybutyrate to cell cultures (Kashiwaya et al., 2000). Moreover, studies performed in transgenic mouse models of AD fed with ketogenic diet showed decreased levels of Aβ aggregates and tau pathology in the brain (Van der Auwera et al., 2005; Kashiwaya et al., 2013). The effects of ketogenic diet on PD have also been investigated. Administration of β-hydroxybutyrate to mice treated with MPTP protected from neurodegeneration and motor impairment (Tieu et al., 2003). Similarly, PD pathology induced by 6-hydroxydopamine in rats was attenuated when a ketogenic diet was administered (Cheng et al., 2009).

Another process that links metabolism with aging and neurodegeneration is the circadian clock. Sleep problems and circadian malfunctions are known consequences of aging and NDs and they are also hallmarks of early stages of NDs (Musiek and Holtzman, 2016). In humans, the control of the circadian rhythm is based on the interaction of CLOCK with ARNTL, a protein found in high levels at the beginning of the day. This complex activates PER and CRY, which is upregulated at night and blocks the CLOCK-ARNTL complex at the start of the night cycle. When CRY levels decrease in the morning, the original complex forms again and re-starts the day cycle 24 h later (Videnovic et al., 2014). This process occurs mostly in neurons from the suprachiasmatic nucleus (SCN), the peacemaker of our body, and the activity of these neurons controls up to 10% of the human genome. Furthermore, degeneration of this group of neurons causes sleep and circadian disruption, which leads to increase in ROS production (Koh et al., 2006; Wang et al., 2012), inflammation (Prolo et al., 2005), proteostasis alterations (Stratmann et al., 2012), and neurodegeneration (Holth et al., 2017). Genzer et al. linked the circadian changes with the levels of BDNF, the most abundant neurotrophin in the brain that causes neurodegeneration when its levels are low. When mice were fed a high fat diet, circadian levels of brain and liver BDNF were altered, mTOR was downregulated and AMPK was activated, which could link circadian clock with obesity and neurodegeneration (Genzer et al., 2016). In *Drosophila*, downregulation of other two modifiers of circadian clock, Spag and Dbt, causes upregulation of the caspase Dronc which cleaves tau and increases neurodegeneration in a model of tauopathy (Means et al., 2015). As in the case of caloric restriction, circadian rhythm is also regulated by Sirtuins and FOXO. Notably, SIRT1 is a master regulator of the circadian clock, activating the key components CLOCK and ARNTL. As the organism ages, SIRT1 decreases in the SCN failing to properly control the circadian clock (Chang and Guarente, 2014). Supporting this mechanism, NAD^+^ levels also follow a rhythm regulated by the circadian levels of NAMPT (nicotinamide phosphoribosyltransferase), an important step in NAD^+^ metabolism (Nakahata et al., 2009). Even though there is no solid connection yet between circadian rhythm and axonal degeneration, it would be interesting to further explore the mentioned components of circadian regulation and NAD^+^ levels on axonal degeneration and its connection to neurodegenerative conditions.

### Macromolecular damage

Several external sources and internal metabolic processes generate as by-product free radicals (FR) such as ROS (Lipsky and King, 2015). For a long time the generation of FR has been linked to aging (Harman, 1956), and the theory that senescence is associated with the accumulation of oxidative damage to macromolecules caused by ROS has been focus of intense research. Damage of organic molecules by FR affects different processes such as proteostasis and response to stress, and importantly puts the mitochondria in a central stage of the aging process. Mitochondria is the largest ROS generator, and in conditions of excessive ROS production, damage to key mitochondrial proteins and DNA occurs, leading to mitochondrial dysfunction, decreased energy production and overall senescence of the cell (Richardson and Schadt, 2014). It has been proposed that with increasing age, the low demand of energy produced by mitochondria due to sedentary lifestyle induces metabolic changes that lead to altered reductants/oxidants ratio. This change triggers a shift favoring an oxidized redox state leading to macromolecular damage (Brewer, 2010). However, whether macromolecular damage caused by oxidative stress constitutes the cause or the consequence of aging and ND-related mechanisms remains yet unclear.

The effect of oxidative damage on macromolecules has been extensively studied in relation to NDs. For example, In the case of ALS, the first genetic link to the disease was made with the discovery of mutations in superoxide dismutase SOD1, an enzyme that catalyzes the conversion of the toxic ${\text{O}}_{2}^{-}$ anions into O_2_ and H_2_O_2_ (Rosen et al., 1993). Oxidative damage in the mitochondria was immediately linked to the disease and further studies aimed at testing potential pharmacological targets in SOD1 transgenic models revealed modest beneficial outcomes (Julien and Kriz, 2006). Furthermore, these studies have been unsuccessfully translated into humans (Ludolph et al., 2009). TDP43 and FUS, other 2 ALS-causative genes, are involved in the prevention or repair of transcription-associated DNA damage, as their depletion increases DNA damage (Hill et al., 2016). Similarly, increased DNA damage is observed in iPSC-derived neurons with expansions in the C9orf72 gene. These cells also show mitochondrial dysfunction and increased oxidative stress (Lopez-Gonzalez et al., 2016). In addition, mitochondrial dysfunction and oxidative macromolecular damage are prominent features of AD. Using primary neuronal cultures from 3xTg-AD mice, Gosh et al. demonstrated an early, reversible oxidized redox state compared to wild-type neurons. This oxidized state preceded an age-related increase in ROS levels and macromolecular ROS damage (Ghosh et al., 2012). Moreover, a proteomic study performed in early AD subjects revealed that lipid peroxidation is an early event in the progression of AD (Reed et al., 2009). Looking at the impact of oxidative damage on the pathology displayed by the AD transgenic model Tg2576, a recent study showed that ROS constitute a key contributor to the development of cerebral amyloid angiopathy, vasomotor dysfunction and microhemorrhage (Han et al., 2015). Similarly, loss of glutathione and increased oxidative DNA and protein damage were observed in an *in vitro* model of PD, where inhibition of mitochondrial complex I by rotenone, induced the typical features of PD including aggregation of α-synuclein (Sherer et al., 2002).

Axonal integrity, which as previously discussed is altered at initial stages of NDs, can be disrupted as consequence of oxidative damage to axonal macromolecules. An *in vitro* study performed in myelin purified from rats showed that myelin-associated protein and lipids are highly vulnerable to oxidative damage (Bongarzone et al., 1995). A recent study in aged wild-type mice demonstrated that motor nerve dysfunction triggered by axonal and myelin damage was associated with a decline in antioxidant defense mechanisms, which led to oxidative protein and lipid damage (Hamilton et al., 2016). Additionally, a number of studies have shown that oxidative damage to axonal components can trigger defects in transport across the axon (Roediger and Armati, 2003; Sharma et al., 2010), which is an early feature of NDs (De Vos et al., 2007; Chu et al., 2012; Sadleir et al., 2016).

## Axonal degeneration at early stages of age-related neurodegenerative conditions

As reviewed above, many of the changes at the molecular and cellular level that occur during the aging process may have an impact on the integrity of axons. Importantly, the evidence indicates that axonal degeneration constitutes an early phase in the process of neurodegeneration that is shared by different age-related neurological diseases (Dadon-Nachum et al., 2010; Adalbert and Coleman, 2013; Tagliaferro and Burke, 2016). In this section we will examine the association of specific NDs with the pillars of aging and evidence implicating axonal degeneration in their pathophysiology. We will focus specifically on AD, PD, and ALS, which constitute the most common age-related NDs. A summary of this information is presented in Tables 1, 2.

**Table 1:** Association of AD, PD and ALS with the pillars of aging.

	**Alzheimer's disease**	**Parkinson's disease**	**Amyotrophic lateral sclerosis**
Proteostasis	IRE1 signaling activation (Duran-Aniotz et al., 2017) Rescue by inhibition of ER acetylation (Peng et al., 2016) Elevated UPR markers (Hoozemans et al., 2005)	Altered autophagy (Li et al., 2017) Proteostasis alterations in stem cells (Chung et al., 2013) ATF6α protection (Egawa et al., 2011)	Decreased foldases and chaperones (Filareti et al., 2017) Mitochondrial UPR ERα activation (Riar et al., 2017) Rescue by XBP1 deficiency (Hetz et al., 2009)
Inflammation	Protection by TNF inhibition (MacPherson et al., 2017) Aβ-associated microglia hyperreactivity (Yin et al., 2017) Role of variant TREM2 (Jonsson et al., 2013)	The role of IL1 (Pott Godoy et al., 2008) Microglial activation (Gerhard et al., 2006) TNFα overexpressed (Mogi et al., 1994)	Inflammation and necroptosis (Ito et al., 2016) Glial activation (Alshikho et al., 2016) Decreased levels of α-1-antitrypsin (Wormser et al., 2016)
Stem cells and regeneration	APP binding to clathrin decreased in NSC (Poulsen et al., 2017) Altered neurogenesis (Unger et al., 2016) Altered stem cell proliferation and neurogenesis (Wu et al., 2016)	α synuclein-induced alteration of neurogenesis (Desplats et al., 2012) Impaired generation of neural precursor cells (Höglinger et al., 2004)	Protective effect of NSC on number and function of motor neurons in SOD1 rats (Xu et al., 2009)
Adaptation to stress	Role of DNA repair factor BRCA1 (Suberbielle et al., 2015) Role of EphB2 depletion (Cissé et al., 2010) Altered glutathione metabolism (Liu et al., 2004)	Altered DNA damage repair (Sepe et al., 2016) Role of XBP1 (Valdes et al., 2014) Altered antioxidant response (Sofic et al., 1992)	Protection by XBP1 deficiency (Hetz et al., 2009) DNA repair dysfunction (Kikuchi et al., 2002) Dysfunction of heat shock response (Chen et al., 2016)
Epigenetics	Role of DNA hydroxymethylation (Zhao et al., 2017) Decreased methylation of CREB regulated transcription coactivator 1 gene (Mendioroz, 2016) DNA methylation near TREM2 (Smith et al., 2016)	α-synuclein involved in histone methylation (Sugeno et al., 2016) Epigenetic deregulation in iPSC-derived dopaminergic neurons (Fernandez-Santiago et al., 2015) Decreased methylation of α-synuclein gene (Jowaed et al., 2010)	Different methylomes in T-cell and monocytes (Lam et al., 2016) Role for cytosine demethylation (Esanov et al., 2016) Rescue by C9orf72 hypermethylation (Liu et al., 2015)
Metabolism	Glucose metabolism (Chiotis et al., 2017) Fatty acid metabolism (Snowden et al., 2017) Metabolites of ornithine (Inoue et al., 2013)	Altered fat distribution (Bernhardt et al., 2016) Changes in glucose metabolism (Dunn et al., 2014) Iron metabolism is altered (Logroscino et al., 1997)	Mitochondrial bioenergetics (Ladd et al., 2017) Astrocyte metabolism (Madji Hounoum et al., 2017) Mutations in transcription-associated DNA damage repair proteins (Hill et al., 2016)
Macro molecular damage	Redox changes (Ghosh et al., 2012) Oxidative DNA damage in leukocytes (Migliore et al., 2005) Peroxynitrite involved in oxidative damage (Smith et al., 1997)	Lipid peroxidation (Mythri et al., 2011) Peripheral protein oxidation (Saito et al., 2009) Mitochondrial impairment and oxidative damage (Sherer et al., 2002)	SOD gene mutations (Rosen et al., 1993) Mutations in iPSC-derived neurons linked with oxidative stress (Lopez-Gonzalez et al., 2016)

**Table 2:** Evidence for axonal degeneration in the pathophysiology of AD, PD and ALS.

**Disease**	**Evidence for axonal degeneration in NDs**	**References**
Alzheimer's disease	Axonal pathology triggered by Aβ precedes cell body death	Adalbert et al., 2009
	Axonal leakage, swollen axons, and varicosities are associated with Aβ plaques and hyperphosphorylated tau in AD brains	Xiao et al., 2011
	Autophagic vesicles are linked with axonal pathology in transgenic AD mice	Sanchez-Varo et al., 2011
	Microtubule-stabilizing agent Epothilone D reduces axonal dysfunction un a mouse model of tau	Zhang et al., 2012
	Aβ oligomers cause microtubule depolymerization leading to altered axonal trafficking	Sadleir et al., 2016
Parkinson's disease	Alterations in axonal transport associated with α-synuclein mutations *in vitro*	Saha, 2004
	Degeneration of axons precedes loss of cell bodies in PD patients	Orimo et al., 2005
	Transgenic α-synuclein mouse model shows striatal dopaminergic axonal, but not cell body, disruption	Tofaris, 2006
	α-synuclein is linked with axonal degeneration which iniciates at the distal axon and continues retrograde	Orimo et al., 2008
	α-synuclein rat model shows altered axonal transport	Chung C. Y. et al., 2009
	Transgenic LRRK2 mouse model shows dopaminergic axonal, but not cell body, disruption	Li et al., 2009
	Axonal pathology triggered by α-synuclein propagates later to the soma is associated with neuronal dysfunction	Volpicelli-Daley et al., 2011
	Early decline in axonal transport associated with α-synuclein aggregation in PD patients	Chu et al., 2012
	Autophagy is involved in axonal pathology and associated with α-synuclein and LRRK2 proteins	Friedman et al., 2012
Amyotrophic lateral sclerosis	Axonal pathology starts at the distal axon and continues in a “dying back” pattern in the innervated muscle fibers	Fischer et al., 2004
	SARM1 gene mutations are linked with ALS development	Fogh et al., 2014
	Defects in axonal transport constitute a typical feature in Drosophila models of ALS	Baldwin et al., 2016
	Axonal degeneration is mediated by necroptosis and inflammation in ALS	Ito et al., 2016
	Potassium channel abnormalities are linked to axon degeneration in ALS mouse model	Maglemose et al., 2017
	ALS-related mutations change the subcellular expression and localization of RNAs within neuronal axon	Rotem et al., 2017

### Alzheimer's disease

AD is a progressive neurodegenerative disorder and constitutes the most frequent form of dementia in the elderly population (Alzheimer's Association, 2016). Although several risk factors have been associated with the pathophysiology of sporadic AD, aging being the most important one, its exact cause remains unrevealed. However, compelling evidence indicate that Aβ dyshomeostasis constitutes a key event involved in the etiopathogenesis of AD, which promotes the accumulation of the protein and further development of all the neuropathological and clinical features of the disease (Selkoe and Hardy, 2016).

As discussed above, perturbation of the proteostasis network leading to the accumulation of protein aggregates is a normal feature of aging, and there is increasing evidence showing that this also occurs in AD (Hoozemans et al., 2005, 2009; Peng et al., 2016; Duran-Aniotz et al., 2017). Hence, it is possible that deterioration of the cellular function as a consequence of aging leads to the imbalance of Aβ production and degradation, triggering the abnormal accumulation of the peptide, reaching toxic levels. Indeed, although Aβ deposition in the brain constitutes the main pathological feature of AD, this process also occurs during normal aging. Histopathological analyses have revealed that in the cases where Aβ deposition is present in brain tissue from non-demented individuals, the amyloid structures are not associated with abnormal neuronal processes, synapse loss, and cognitive alterations as occur in AD brain tissue (Serrano-Pozo et al., 2011), suggesting that degeneration of axons and dendrites (also referred to as neurites) constitutes a key event involved in the Aβ-related mechanisms that participate in the transition from normal aging to dementia. Moreover, neurons affected in AD follow a dying-back pattern of degeneration, where axonal disruption and synaptic loss precede neuronal cell death and manifest in early stages of the disease (Serrano-Pozo et al., 2011; Adalbert and Coleman, 2013).

Although Aβ-related axonal and dendritic dystrophy is an early histopathological observation in postmortem AD brains (Knowles et al., 1999; Nelson et al., 2012) and in animal models of AD (Mucke et al., 2000), whether Aβ deposition is the cause of axonal and dendritic degeneration or constitutes a consequence of an underlying neurodegenerative process remains currently unknown and there is evidence demonstrating that both possibilities may occur. Traumatic brain injury (TBI) is one of the main environmental risk factors for the development of AD (Alzheimer's Association, 2016). Diffuse axonal injury represents a typical consequence of TBI, where disruption of the cytoskeleton results in swollen axons, altered axonal transport, and mitochondrial dysfunction (Choe, 2016). Intra-axonal upregulation of the amyloid precursor protein (APP) and increased processing of the protein resulting in diffuse Aβ plaque deposition occurs quickly after TBI and notably, studies in long-term survivors have revealed that axonal degeneration persists and mature Aβ plaques and tau pathology develops, which is associated with cognitive impairment (Johnson et al., 2011). These studies suggest that Aβ accumulation occurs due to the disruption of axonal integrity following injury. Hence, it is possible that the axonal damage that occurs as a consequence of aging, could also contribute to the accumulation of Aβ. In a recent study, looking at the mechanisms of dystrophic neurite formation in AD, Sadleir and colleagues were able to show that exposure of cultured neurons to Aβ oligomers caused microtubule depolymerization leading to altered axonal trafficking (Sadleir et al., 2016). The results were confirmed in brain tissue from both AD patients and transgenic mice, where they observed that dystrophic neurites in the close proximity to Aβ plaques contained low microtubule density, accumulation of autophagic intermediates and increased β-site APP cleaving enzyme (BACE1) and APP levels, which caused enhanced generation of Aβ (Sadleir et al., 2016).

Extensive research focusing on the mechanisms underlying Aβ neurotoxicity has been undertaken and as a result, its association with numerous pathogenic pathways has been suggested. Among the pathways activated by Aβ, most of them are implicated in the process of axonal degeneration, including oxidative stress (Behl et al., 1994; Hensley et al., 1994; Butterfield et al., 2013), mitochondrial dysfunction (Devi, 2006; Wang et al., 2009; Kerr et al., 2017), and abnormal calcium signaling (Mattson et al., 1992; Furukawa et al., 1996; Demuro et al., 2005; Meyer-Luehmann et al., 2008). To date, AD drug development has been based primarily on the amyloid hypothesis, and the majority of randomized controlled trials have been designed to target this protein. However, the overall outcomes have been dramatic, with a 99.6% failure rate for approval (Cummings et al., 2014). Nevertheless, the trails have arguably been conducted late and when there is extensive pathology and degeneration. As mentioned above, axonal degeneration represents an early event during the progression of AD, hence, unveiling the exact mechanisms that trigger the degeneration of axons represent a promising step in the field of AD drug development.

### Parkinson's disease

PD is the most common motor-related ND. The precise cause of the disease remains largely unknown; although it is thought that PD is the result of a combination of genetic and environmental risk factors (Pires et al., 2017). Due to the strong association between mutations in α-synuclein gene and the development of familial PD, a central focus of PD research has been the misfolding and deposition of this protein (Gao et al., 2008; Imaizumi et al., 2012; Gonzalez-Horta, 2015; Wang and Hay, 2015).

Recent evidence indicate that the degeneration of axons of dopaminergic neurons constitute an early event in PD development. Thus, axonal degeneration plays a critical yet unclear role in this disease. Following the observation that degeneration of the distal axons of the cardiac sympathetic nerve precedes loss of cell bodies in PD patients (Orimo et al., 2005), Orimo and colleagues focused on the involvement of α-synuclein on the degeneration of axons and the timing of this process and demonstrated that the pathology commence at the distal axon and continues in a retrograde fashion (Orimo et al., 2008). Additional evidence supporting these findings includes *in vitro* studies of primary neurons exposed to α-synuclein fibrils, where recruitment of endogenous α-synuclein to form insoluble aggregates was observed. Interestingly, the pathology was initially observed in axons and was associated to neuronal dysfunction, propagating proximally to the soma leading to neuronal cell death (Volpicelli-Daley et al., 2011). Moreover, studies in a transgenic mouse model expressing a mutant form of α-synuclein revealed striatal dopaminergic axonal disruption, while the integrity of cell bodies of dopaminergic neurons was maintained (Tofaris, 2006). Similarly, characterization of a transgenic mouse model that expresses mutant leucine-rich repeat kinase 2 (LRRK2), the single most common cause of inherited PD, showed a significant alteration of axonal integrity, however, no loss of dopaminergic neurons was observed (Li et al., 2009).

Based on the evidence indicating that axons are the first site of degenerative change and are compromised before cell soma, Chu and colleagues used human PD tissue to investigate axonal transport in the initial stages of PD. The group demonstrated an early decline in axonal transport motor proteins, which occurred before the alteration of dopaminergic phenotypic markers and was associated with α-synuclein aggregation (Chu et al., 2012). This result is in line with previous studies demonstrating alterations in axonal transport associated with α-synuclein mutations *in vitro* (Saha, 2004) and *in vivo* (Chung C. Y. et al., 2009). Looking at the mechanisms of axonal degeneration in PD, a study performed in an autophagy-deficient mouse model revealed that deletion of the autophagy gene Atg7 triggered early dendritic and axonal dystrophy, which was associated with enhanced levels of endogenous α-synuclein and LRRK2 proteins. This study suggests that alterations in autophagy might be involved in the pathogenesis of sporadic PD and linked with axonal degeneration (Friedman et al., 2012).

A workshop presenting the state-of-the-art of axonal pathology research in PD was recently carried out and a summary of the current knowledge on the field was published (Kurowska et al., 2016). The view of axonal degeneration as an early process in the development of PD is discussed and the importance of finding early diagnostic markers, as well as targeting axonal degeneration as a preventive measure are highlighted.

### Amyotrophic lateral sclerosis

ALS is a progressive adult-onset disorder, characterized by the selective death of upper and lower motor neurons leading to paralysis and muscle atrophy. About 10% of ALS cases have a genetic cause, and many different forms of the disease are the result of different genetic mutations (Al-Chalabi et al., 2016). Currently, the cause of the sporadic form of the disease remains unknown (Pasinelli and Brown, 2006).

An important component of the neuronal dysfunction in ALS is the degeneration of axons (Ferraiuolo et al., 2011). The disease initiates at the distal motor axons and continues in a “dying back” pattern, with denervation and reinnervation taking place in the innervated muscle fibers at early stages (Fischer et al., 2004). Evidence suggest that in ALS, axonal damage takes place before loss of cell bodies and the onset of clinical symptoms, which appear only after a large proportion of motor units are lost (Dadon-Nachum et al., 2010). Interestingly, the Wld^S^ mouse model shows delayed axon degeneration in some peripheral neuropathies. However, it has not been successful on improving the symptoms and axonal pathology present in SOD1 mutant rodent models of ALS (Vande Velde et al., 2004; Fischer et al., 2005). Nonetheless, by performing a genome-wide association meta-analysis, Fogh et al. identified the SARM1 locus as spot for three SNPs linked to patients with ALS (Fogh et al., 2014).

Several studies have shown impaired axonal transport in ALS, which has been demonstrated to constitute an early event during the progression of the disease (Williamson and Cleveland, 1999; Murakami et al., 2001; De Vos et al., 2007). By instance, altered mitochondrial transport through the axon was demonstrated in two different SOD1 mutant mouse models of ALS (Magrané et al., 2014). Furthermore, mutations in the RNA-binding protein TDP-43 can cause ALS and interestingly, it was recently demonstrated that this protein functions as an mRNA transporter across the axonal cytoskeleton and that mutations in this protein leading to ALS, alter this transport function (Alami et al., 2014). Notably, genetic studies performed in families with ALS revealed mutations in the genes encoding the transporter proteins dynactin (Puls et al., 2003) and tubulin (Smith et al., 2014) which lead to reduced binding of the mutant protein to microtubules and decreased repolymerization capability respectively.

As discussed previously, sirtuins are an important link between aging and neurodegeneration. In ALS, altered sirtuin levels have been observed in both transgenic mouse models (Han et al., 2012) and patients tissue (Körner et al., 2013). Moreover, in *Drosophila*, Sirtuin1 was described as a suppressor of neurodegeneration in the ALS model DVAP-P58S (Sanhueza et al., 2015). Sirtuin 1 protection from axonal degeneration is regulated by Nmnat1, the protein overexpressed in the WldS model (Araki et al., 2004). Furthermore, resveratrol, a polyphenol that exhibits beneficial effects in NDs, protects against WD by activating Sirtuin 1 through dissociation from its inhibitor DBC1 (Calliari et al., 2014). There is evidence indicating that the metabolites obtained from NADH play a crucial role on the effect of Sirtuins (Jasper, 2013), and considering that these metabolites, especially nicotinamide mononucleotide (NMN), are crucial in the axonal degenerative process (Di Stefano et al., 2017), it seems plausible that sirtuins and NAD^+^ metabolism might play a central role on the ALS pathomechanism, even as a potential therapeutic target for the disease (Pasinetti et al., 2013; Tang, 2016).

As mentioned previously, different gene mutations can lead to several forms of ALS. Together, the evidence presented above suggests that axonal degeneration plays a key role in ALS pathophysiology and that common mechanisms involved in axonal pathology can contribute to the development of the different forms of ALS, which will benefit the search for potential therapies to tackle them before the disease is already declared and irreversible.

### Huntington's disease and other pathologies

HD is a disease that in contrast to the previously described ones, it is the most common monogenic neurological disease. HD is caused by the expansion of the gene codifying for the huntingtin protein. The mutant version carries long polyglutamine sequences encoded by repeated CAG. In HD patients, expression of mutant Htt affects neurons in the striatum and cortex, triggering neuronal dysfunction and apoptosis. Neuronal death causes motor and cognitive impairment, leading to death of patients 18 years after the onset of motor problems (Bates et al., 2015).

Abnormal splicing and formation of amino-terminal Htt fragments are consequences in which the translation of mutant huntingtin causes toxicity in the disease. Htt fragments aggregate in the nucleus and sequester other proteins disrupting the proteostasis network. Interestingly, Htt fragments also affect mitochondrial function and cellular trafficking, leading to axonal dysfunction and degeneration. Even though experimental evidence demonstrate that axonal degeneration is an early event in HD (Li and Conforti, 2013), its temporal relation with cell loss and disease symptoms is not completely understood. It has been determined in mouse models of HD and human patients that callosal axons degenerate long before the onset of motor symptoms (Gatto et al., 2015). This phenotype was worsened with age and suggests a dying-back pattern of degeneration in HD (Gatto et al., 2015). Early signs of axonal aggregates were also described in striatal axonal projections to the globus pallidus and the substantia nigra of mice expressing full-length mutant huntingtin. Neuropil aggregates were associated with degenerated mitochondria and with defects on protein transport (Li et al., 2001). Importantly, a different study determined that the axonal swellings were formed age-dependently and were independent of inclusions in the soma, suggesting that axon degeneration precedes death of other neuronal compartments in a model of HD (Marangoni et al., 2014). Mechanistically, axonal pathology in an HD mouse model shares molecular pathways with a model of axonal injury, as proteomic screens identified proteins with similar expression levels in both models (Wishart et al., 2012). Transport failure and the associated axonal dysfunction therefore appear as central causes of early HD symptoms.

There are other age-related NDs where axonal dysfunction plays a central role, including glaucoma (Weinreb et al., 2016), progressive supranuclear palsy (Lopez et al., 2016), and vascular dementia (Elahi and Miller, 2017). They share commonalities with the disorders already covered here, and importantly, some of the overlapping symptoms in patients can be caused by accumulation of axonal dysfunction with age, regardless the specific causative gene of each disorder.

## Concluding remarks

Why some individuals develop neurodegeneration and associated cognitive decline with advanced age, while others are able to preserve the cognitive function, has been the focus of intense research in recent years. However, the exact age-related molecular and cellular changes that trigger this susceptibility to neurodegeneration remain to be established. Here, we revised the evidence that support the potential role of axonal degeneration in this transition. As discussed in this review, many molecular and cellular changes that occur as organisms age may contribute to the deterioration of axons. Notably, increasing evidence in recent years has raised the awareness of axonal pathology as an early, common contributor to the pathomechanism of different age-related neurological diseases. This pathological overlapping shared by NDs represents an important focus of research not only for the impact in our current understanding of the etiology of this diseases, but also for the drug development field as it might provide potential targets for future therapeutic and, most importantly, preventative strategies aimed at limiting axonal and therefore neuronal degeneration in NDs.

## Author contributions

NS, MS planned, researched and wrote the manuscript. PM, FC edited and helped in the planning of the manuscript.

### Conflict of interest statement

The authors declare that the research was conducted in the absence of any commercial or financial relationships that could be construed as a potential conflict of interest.

## References

[B1] Adalbert R. Coleman M. P. (2013). Review: axon pathology in age-related neurodegenerative disorders. Neuropathol. Appl. Neurobiol. 39, 90–108. 10.1111/j.1365-2990.2012.01308.x 23046254

[B2] Adalbert R. Nogradi A. Babetto E. Janeckova L. Walker S. A. Kerschensteiner M.. (2009). Severely dystrophic axons at amyloid plaques remain continuous and connected to viable cell bodies. Brain 132, 402–416. 10.1093/brain/awn312 19059977

[B3] Alami N. H. Smith R. B. Carrasco M. A. Williams L. A. Winborn C. S. Han S. S. W.. (2014). Axonal transport of TDP-43 mRNA granules is impaired by ALS-causing mutations. Neuron 81, 536–543. 10.1016/j.neuron.2013.12.018 24507191 PMC3939050

[B4] Al-Chalabi A. van den Berg L. H. Veldink J. (2016). Gene discovery in amyotrophic lateral sclerosis: implications for clinical management. Nat. Rev. Neurol. 13, 96–104. 10.1038/nrneurol.2016.182 27982040

[B5] Alshikho M. J. Zürcher N. R. Loggia M. L. Cernasov P. Chonde D. B. Izquierdo Garcia D.. (2016). Glial activation colocalizes with structural abnormalities in amyotrophic lateral sclerosis. Neurology 87, 2554–2561. 10.1212/WNL.0000000000003427 27837005 PMC5207001

[B6] Alzheimer's Association (2016). 2016 Alzheimer's disease facts and figures. Alzheimers Dement. 12, 459–509. 10.1016/j.jalz.2016.03.001 27570871

[B7] Araki T. Sasaki Y. Milbrandt J. (2004). Increased nuclear NAD biosynthesis and SIRT1 activation prevent axonal degeneration. Science 305, 1010–1013. 10.1126/science.1098014 15310905

[B8] Ash C. E. Merry B. J. (2011). The molecular basis by which dietary restricted feeding reduces mitochondrial reactive oxygen species generation. Mech. Ageing Dev. 132, 43–54. 10.1016/j.mad.2010.12.001 21172374

[B9] Avery M. A. Sheehan A. E. Kerr K. S. Wang J. Freeman M. R. (2009). Wld Srequires Nmnat1 enzymatic activity and N16–VCP interactions to suppress Wallerian degeneration. J. Cell Biol. 184, 501–513. 10.1083/jcb.200808042 19237597 PMC2654119

[B10] Ayyaz A. Jasper H. (2013). Intestinal inflammation and stem cell homeostasis in aging *Drosophila melanogaster*. Front. Cell. Infect. Microbiol. 3:98. 10.3389/fcimb.2013.00098 24380076 PMC3863754

[B11] Baldwin K. R. Godena V. K. Hewitt V. L. Whitworth A. J. (2016). Axonal transport defects are a common phenotype in Drosophila models of ALS. Hum. Mol. Genet. 15, 2378–2392. 10.1093/hmg/ddw105 PMC5181624 27056981

[B12] Barrientos S. A. Martinez N. W. Yoo S. Jara J. S. Zamorano S. Hetz C.. (2011). Axonal degeneration is mediated by the mitochondrial permeability transition pore. J. Neurosci. 31, 966–978. 10.1523/JNEUROSCI.4065-10.2011 21248121 PMC3245862

[B13] Bates G. P. Dorsey R. Gusella J. F. Hayden M. R. Kay C. Leavitt B. R. (2015). Huntington disease. Nat. Rev. Dis. Primers 26, 15005–15021. 10.1038/nrdp.2015.5 27188817

[B14] Behl C. Davis J. B. Lesley R. Schubert D. (1994). Hydrogen peroxide mediates amyloid beta protein toxicity. Cell 77, 817–827. 10.1016/0092-8674(94)90131-7 8004671

[B15] Belzil V. V. Bauer P. O. Gendron T. F. Murray M. E. Dickson D. Petrucelli L. (2014). Characterization of DNA hypermethylation in the cerebellum of c9FTD/ALS patients. Brain Res. 1584, 15–21. 10.1016/j.brainres.2014.02.015 24530272 PMC4130919

[B16] Bernhardt D. Muller H.-P. Ludolph A. C. Dupuis L. Kassubek J. (2016). Body fat distribution in Parkinson's disease: an MRI-based body fat quantification study. Parkinsonism Relat. Disord. 33, 84–89. 10.1016/j.parkreldis.2016.09.016 27665038

[B17] Blurton-Jones M. Spencer B. Michael S. Castello N. A. Agazaryan A. A. Davis J. L.. (2014). Neural stem cells genetically-modified to express neprilysin reduce pathology in Alzheimer transgenic models. Stem Cell Res. Ther. 5:46. 10.1186/scrt440 25022790 PMC4055090

[B18] Bongarzone E. R. Pasquini J. M. Soto E. F. (1995). Oxidative damage to proteins and lipids of CNS myelin produced by *in vitro* generated reactive oxygen species. J. Neurosci. Res. 41, 213–221. 10.1002/jnr.490410209 7650757

[B19] Boyd-Kirkup J. D. Green C. D. Wu G. Wang D. Han J.-D. J. (2013). Epigenomics and the regulation of aging. Epigenomics 5, 205–227. 10.2217/epi.13.5 23566097

[B20] Brehme M. Voisine C. Rolland T. Wachi S. Soper J. H. Zhu Y.. (2014). A chaperome subnetwork safeguards proteostasis in aging and neurodegenerative disease. Cell Rep. 9, 1135–1150. 10.1016/j.celrep.2014.09.042 25437566 PMC4255334

[B21] Brewer G. J. (2010). Epigenetic oxidative redox shift (EORS) theory of aging unifies the free radical and insulin signaling theories. Exp. Gerontol. 45, 173–179. 10.1016/j.exger.2009.11.007 19945522 PMC2826600

[B22] Bulteau A.-L. Szweda L. I. Friguet B. (2002). Age-dependent declines in proteasome activity in the heart. Arch. Biochem. Biophys. 397, 298–304. 10.1006/abbi.2001.2663 11795886

[B23] Burnett C. Valentini S. Cabreiro F. Goss M. Somogyvári M. Piper M. D.. (2011). Absence of effects of Sir2 overexpression on lifespan in *C. elegans* and Drosophila. Nature 477, 482–485. 10.1038/nature10296 21938067 PMC3188402

[B24] Butterfield D. A. Swomley A. M. Sultana R. (2013). Amyloid β-peptide (1–42)-induced oxidative stress in Alzheimer Disease: importance in disease pathogenesis and progression. Antioxid. Redox Signal. 19, 823–835. 10.1089/ars.2012.5027 23249141 PMC3749710

[B25] Calixto A. Jara J. S. Court F. A. (2012). Diapause formation and downregulation of insulin-like signaling via DAF-16/FOXO delays axonal degeneration and neuronal loss. PLoS Genet. 8:e1003141. 10.1371/journal.pgen.1003141 23300463 PMC3531479

[B26] Calliari A. Bobba N. Escande C. Chini E. N. (2014). Resveratrol delays Wallerian degeneration in a NAD^+^ and DBC1 dependent manner. Exp. Neurol. 251, 91–100. 10.1016/j.expneurol.2013.11.013 24252177

[B27] Carson M. J. Doose J. M. Melchior B. Schmid C. D. Ploix C. C. (2006). CNS immune privilege: hiding in plain sight. Immunol. Rev. 213, 48–65. 10.1111/j.1600-065X.2006.00441.x 16972896 PMC2633103

[B28] Carty M. Goodbody R. Schroder M. Stack J. Moynagh P. N. Bowie A. G. (2006). The human adaptor SARM negatively regulates adaptor protein TRIF-dependent Toll-like receptor signaling. Nat. Immunol. 7, 1074–1081. 10.1038/ni1382 16964262

[B29] Chang H.-C. Guarente L. (2014). SIRT1 and other sirtuins in metabolism. Trends Endocrinol. Metab. 25, 138–145. 10.1016/j.tem.2013.12.001 24388149 PMC3943707

[B30] Chen C.-Y. Lin C.-W. Chang C.-Y. Jiang S.-T. Hsueh Y.-P. (2011). Sarm1, a negative regulator of innate immunity, interacts with syndecan-2 and regulates neuronal morphology. J. Cell Biol. 193, 769–784. 10.1083/jcb.201008050 21555464 PMC3166868

[B31] Chen L. Nye D. M. Stone M. C. Weiner A. T. Gheres K. W. Xiong X.. (2016). Mitochondria and caspases tune nmnat-mediated stabilization to promote axon regeneration. PLoS Genet. 12:e1006503. 10.1371/journal.pgen.1006503 27923046 PMC5173288

[B32] Cheng B. Yang X. An L. Gao B. Liu X. Liu S. (2009). Ketogenic diet protects dopaminergic neurons against 6-OHDA neurotoxicity via up-regulating glutathione in a rat model of Parkinson's disease. Brain Res. 1286, 25–31. 10.1016/j.brainres.2009.06.060 19559687

[B33] Cheng H. C. Kim S. R. Oo T. F. Kareva T. Yarygina O. Rzhetskaya M.. (2011). Akt suppresses retrograde degeneration of dopaminergic axons by inhibition of macroautophagy. J. Neurosci. 31, 2125–2135. 10.1523/JNEUROSCI.5519-10.2011 21307249 PMC3075005

[B34] Chiotis K. Saint-Aubert L. Rodriguez-Vieitez E. Leuzy A. Almkvist O. Savitcheva I.. (2017). Longitudinal changes of tau PET imaging in relation to hypometabolism in prodromal and Alzheimer's disease dementia. Mol. Psychiatry. [Epub ahead of print]. 10.1038/mp.2017.108 28507319

[B35] Choe M. C. (2016). The pathophysiology of concussion. Curr. Pain Headache Rep. 20:42 10.1007/s11916-016-0573-9 27184060

[B36] Chondrogianni N. Georgila K. Kourtis N. Tavernarakis N. Gonos E. S. (2015). 20S proteasome activation promotes life span extension and resistance to proteotoxicity in *Caenorhabditis elegans*. FASEB J. 29, 611–622. 10.1096/fj.14-252189 25395451 PMC4314225

[B37] Christensen B. C. Houseman E. A. Marsit C. J. Zheng S. Wrensch M. R. Wiemels J. L.. (2009). Aging and environmental exposures alter tissue-specific DNA methylation dependent upon CpG island context. PLoS Genet. 5:e1000602. 10.1371/journal.pgen.1000602 19680444 PMC2718614

[B38] Chu Y. Morfini G. A. Langhamer L. B. He Y. Brady S. T. Kordower J. H. (2012). Alterations in axonal transport motor proteins in sporadic and experimental Parkinson's disease. Brain 135, 2058–2073. 10.1093/brain/aws133 22719003 PMC4571141

[B39] Chung C. Y. Khurana V. Auluck P. K. Tardiff D. F. Mazzulli J. R. Soldner F.. (2013). Identification and rescue of α-synuclein toxicity in Parkinson patient-derived neurons. Science 342, 983–987. 10.1126/science.1245296 24158904 PMC4022187

[B40] Chung C. Y. Koprich J. B. Siddiqi H. Isacson O. (2009). Dynamic changes in presynaptic and axonal transport proteins combined with striatal neuroinflammation precede dopaminergic neuronal loss in a rat model of AAV -synucleinopathy. J. Neurosci. 29, 3365–3373. 10.1523/JNEUROSCI.5427-08.2009 19295143 PMC2693917

[B41] Chung H. Y. Cesari M. Anton S. Marzetti E. Giovannini S. Seo A. Y.. (2009). Molecular inflammation: underpinnings of aging and age-related diseases. Ageing Res. Rev. 8, 18–30. 10.1016/j.arr.2008.07.002 18692159 PMC3782993

[B42] Cicchetti F. Saporta S. Hauser R. A. Parent M. Saint-Pierre M. Sanberg P. R. (2009). Neural transplants in patients with Huntington's disease undergo disease-like neuronal degeneration. Proc. Natl. Acad. Sci. U.S.A. 106, 12483–12488. 10.1073/pnas.0904239106 19620721 PMC2713393

[B43] Cissé M. Duplan E. Lorivel T. Dunys J. Bauer C. Meckler X. (2016). The transcription factor XBP1s restores hippocampal synaptic plasticity and memory by control of the Kalirin-7 pathway in Alzheimer model. Mol. Psychiatry. 11:385 10.1038/mp.2016.152 PMC5658671 27646263

[B44] Cissé M. Halabisky B. Harris J. Devidze N. Dubal D. B. Sun B.. (2010). Reversing EphB2 depletion rescues cognitive functions in Alzheimer model. Nature 469, 47–52. 10.1038/nature09635 21113149 PMC3030448

[B45] Coleman M. (2005). Axon degeneration mechanisms: commonality amid diversity. Nat. Rev. Neurosci. 6, 889–898. 10.1038/nrn1788 16224497

[B46] Coleman M. P. Freeman M. R. (2010). Wallerian Degeneration, Wld, S., and Nmnat. Annu. Rev. Neurosci. 33, 245–267. 10.1146/annurev-neuro-060909-153248 20345246 PMC5223592

[B47] Cooper A. A. Gitler A. D. Cashikar A. Haynes C. M. Hill K. J. Bhullar B.. (2006). Alpha-synuclein blocks ER-golgi traffic and Rab1 rescues neuron loss in Parkinson's Models. Science 313, 324–328. 10.1126/science.1129462 16794039 PMC1983366

[B48] Court F. A. Coleman M. P. (2012). Mitochondria as a central sensor for axonal degenerative stimuli. Trends Neurosci. 35, 364–372. 10.1016/j.tins.2012.04.001 22578891

[B49] Cribbs D. H. Berchtold N. C. Perreau V. Coleman P. D. Rogers J. Tenner A. J. (2012). Extensive innate immune gene activation accompanies brain aging, increasing vulnerability to cognitive decline and neurodegeneration: a microarray study. J. Neuroinflammation 9:179 10.1186/1742-2094-9-179 22824372 PMC3419089

[B50] Crook-McMahon H. M. Oláhová M. Button E. L. Winter J. J. Veal E. A. (2014). Genome-wide screening identifies new genes required for stress-induced phase 2 detoxification gene expression in animals. BMC Biol. 12:64 10.1186/s12915-014-0064-6 PMC4160539 25204677

[B51] Cuervo A. M. Dice J. F. (2000). Age-related decline in chaperone-mediated autophagy. J. Biol. Chem. 275, 31505–31513. 10.1074/jbc.M002102200 10806201

[B52] Cummings J. L. Morstorf T. Zhong K. (2014). Alzheimer's disease drug-development pipeline: few candidates, frequent failures. Alzheimers Res. Ther. 6:37. 10.1186/alzrt269 25024750 PMC4095696

[B53] Dadon-Nachum M. Melamed E. Offen D. (2010). The “Dying-Back” phenomenon of motor neurons in ALS. J. Mol. Neurosci. 43, 470–477. 10.1007/s12031-010-9467-1 21057983

[B54] Dasuri K. Zhang L. Ebenezer P. Liu Y. Fernandez-Kim S. O. Keller J. N. (2009). Aging and dietary restriction alter proteasome biogenesis and composition in the brain and liver. Mech. Ageing Dev. 130, 777–783. 10.1016/j.mad.2009.10.003 19896962 PMC2942759

[B55] De Vos K. J. Chapman A. L. Tennant M. E. Manser C. Tudor E. L. Lau K. F.. (2007). Familial amyotrophic lateral sclerosis-linked SOD1 mutants perturb fast axonal transport to reduce axonal mitochondria content. Hum. Mol. Genet. 16, 2720–2728. 10.1093/hmg/ddm226 17725983 PMC4516806

[B56] Deckwerth T. L. Johnson E. M. (1994). Neurites can remain viable after destruction of the neuronal soma by programmed cell death (Apoptosis). Dev. Biol. 165, 63–72. 10.1006/dbio.1994.1234 8088451

[B57] Demuro A. Mina E. Kayed R. Milton S. C. Parker I. Glabe C. G. (2005). Calcium dysregulation and membrane disruption as a ubiquitous neurotoxic mechanism of soluble amyloid oligomers. J. Biol. Chem. 280, 17294–17300. 10.1074/jbc.M500997200 15722360

[B58] Deng H. Gerencser A. A. Jasper H. (2015). Signal integration by Ca^2+^ regulates intestinal stem-cell activity. Nature 528, 212–217. 10.1038/nature16170 26633624 PMC4669953

[B59] Desplats P. Spencer B. Crews L. Pathel P. Morvinski-Friedmann D. Kosberg K. (2012). α-synuclein induces alterations in adult neurogenesis in parkinson disease models via p53-mediated repression of notch1. J. Biol. Chem. 287, 31691–31702. 10.1074/jbc.M112.354522 22833673 PMC3442504

[B60] Devi L. (2006). Accumulation of amyloid precursor protein in the mitochondrial import channels of human Alzheimer's Disease brain is associated with mitochondrial dysfunction. J. Neurosci. 26, 9057–9068. 10.1523/JNEUROSCI.1469-06.2006 16943564 PMC6675337

[B61] Di Stefano M. Loreto A. Orsomando G. Mori V. Zamporlini F. Hulse R. P. (2017). NMN deamidase delays wallerian degeneration and rescues axonal defects caused by NMNAT2 deficiency *in vivo*. Curr. Biol. 27, 784–794. 10.1016/j.cub.2017.01.070 28262487

[B62] Druzhyna N. M. Wilson G. L. LeDoux S. P. (2008). Mitochondrial DNA repair in aging and disease. Mech. Ageing Dev. 129, 383–390. 10.1016/j.mad.2008.03.002 18417187 PMC2666190

[B63] Du H. Guo L. Fang F. Chen D. Sosunov A. A. McKhann G. M.. (2008). Cyclophilin D deficiency attenuates mitochondrial and neuronal perturbation and ameliorates learning and memory in Alzheimer's disease. Nat. Med. 14, 1097–1105. 10.1038/nm.1868 18806802 PMC2789841

[B64] Duan W. Guo Z. Jiang H. Ware M. Li X.-J. Mattson M. P. (2003). Dietary restriction normalizes glucose metabolism and BDNF levels, slows disease progression, and increases survival in huntingtin mutant mice. Proc. Natl. Acad. Sci. U.S.A. 100, 2911–2916. 10.1073/pnas.0536856100 12589027 PMC151440

[B65] Dunn L. Allen G. F. Mamais A. Ling H. Li A. Duberley K. E.. (2014). Dysregulation of glucose metabolism is an early event in sporadic Parkinson's disease. Neurobiol. Aging 35, 1111–1115. 10.1016/j.neurobiolaging.2013.11.001 24300239 PMC3969149

[B66] Duran-Aniotz C. Cornejo V. H. Espinoza S. Ardiles Á. O. Medinas D. B. Salazar C.. (2017). IRE1 signaling exacerbates Alzheimer's disease pathogenesis. Acta Neuropathol. [Epub ahead of print]. 10.1007/s00401-017-1694-x 28341998

[B67] Egawa N. Yamamoto K. Inoue H. Hikawa R. Nishi K. Mori K. (2011). The endoplasmic reticulum stress sensor, ATF6, protects against neurotoxin-induced dopaminergic neuronal death. J. Biol. Chem. 286, 7947–7957. 10.1074/jbc.M110.156430 21131360 PMC3048681

[B68] Elahi F. M. Miller B. L. (2017). A clinicopathological approach to the diagnosis of dementia. Nat. Rev. Neurol. 13, 457–476. 10.1038/nrneurol.2017.96 28708131 PMC5771416

[B69] Esanov R. Belle K. C. van Blitterswijk M. Belzil V. V. Rademakers R. Dickson D. W.. (2016). C9orf72 promoter hypermethylation is reduced while hydroxymethylation is acquired during reprogramming of ALS patient cells. Exp. Neurol. 277, 171–177. 10.1016/j.expneurol.2015.12.022 26746986 PMC4761318

[B70] Essuman K. Summers D. W. Sasaki Y. Mao X. DiAntonio A. Milbrandt J. (2017). The SARM1 toll/interleukin-1 receptor domain possesses intrinsic NAD+ cleavage activity that promotes pathological axonal degeneration. Neuron 93, 1334.e5–1343.e5. 10.1016/j.neuron.2017.02.022 28334607 PMC6284238

[B71] Fairbairn N. G. Meppelink A. M. Ng-Glazier J. Randolph M. A. Winograd J. M. (2015). Augmenting peripheral nerve regeneration using stem cells: A review of current opinion. World J. Stem Cells 7, 11–26. 10.4252/wjsc.v7.i1.11 25621102 PMC4300921

[B72] Fang Y. Soares L. Teng X. Geary M. Bonini N. M. (2012). A novel Drosophila model of nerve injury reveals an essential role of nmnat in maintaining axonal integrity. Curr. Biol. 22, 590–595. 10.1016/j.cub.2012.01.065 22425156 PMC3347919

[B73] Fargnoli J. Kunisada T. Fornace A. J. Schneider E. L. Holbrook N. J. (1990). Decreased expression of heat shock protein 70 mRNA and protein after heat treatment in cells of aged rats. Proc. Natl. Acad. Sci. U.S.A. 87, 846–850. 10.1073/pnas.87.2.846 2300568 PMC53363

[B74] Fernandez-Santiago R. Carballo-Carbajal I. Castellano G. Torrent R. Richaud Y. Sanchez-Danes A.. (2015). Aberrant epigenome in iPSC-derived dopaminergic neurons from Parkinson's disease patients. EMBO Mol. Med. 7, 1529–1546. 10.15252/emmm.201505439 26516212 PMC4693505

[B75] Ferraiuolo L. Kirby J. Grierson A. J. Sendtner M. Shaw P. J. (2011). Molecular pathways of motor neuron injury in amyotrophic lateral sclerosis. Nat. Rev. Neurol. 7, 616–630. 10.1038/nrneurol.2011.152 22051914

[B76] Ferrington D. A. (2005). Altered proteasome structure, function, and oxidation in aged muscle. FASEB J. 19, 644–646. 10.1096/fj.04-2578fje 15677694

[B77] Ferron S. R. Marques-Torrejon M. A. Mira H. Flores I. Taylor K. Blasco M. A.. (2009). Telomere shortening in neural stem cells disrupts neuronal differentiation and neuritogenesis. J. Neurosci. 29, 14394–14407. 10.1523/JNEUROSCI.3836-09.2009 19923274 PMC6665809

[B78] Filareti M. Luotti S. Pasetto L. Pignataro M. Paolella K. Messina P.. (2017). Decreased levels of foldase and chaperone proteins are associated with an early-onset amyotrophic lateral sclerosis. Front. Mol. Neurosci. 10:99. 10.3389/fnmol.2017.00099 28428745 PMC5382314

[B79] Fischer L. R. Culver D. G. Davis A. A. Tennant P. Wang M. Coleman M.. (2005). The WldS gene modestly prolongs survival in the SOD1G93A fALS mouse. Neurobiol. Dis. 19, 293–300. 10.1016/j.nbd.2005.01.008 15837585

[B80] Fischer L. R. Culver D. G. Tennant P. Davis A. A. Wang M. Castellano-Sanchez A.. (2004). Amyotrophic lateral sclerosis is a distal axonopathy: evidence in mice and man. Exp. Neurol. 185, 232–240. 10.1016/j.expneurol.2003.10.004 14736504

[B81] Fogh I. Ratti A. Gellera C. Lin K. Tiloca C. Moskvina V.. (2014). A genome-wide association meta-analysis identifies a novel locus at 17q11.2 associated with sporadic amyotrophic lateral sclerosis. Hum. Mol. Genet. 23, 2220–2231. 10.1093/hmg/ddt587 24256812 PMC3959809

[B82] Fredriksson Å. Johansson Krogh E. Hernebring M. Pettersson E. Javadi A. Almstedt A.. (2012). Effects of aging and reproduction on protein quality control in soma and gametes of *Drosophila melanogaster*. Aging Cell 11, 634–643. 10.1111/j.1474-9726.2012.00823.x 22507075

[B83] Friedman L. G. Lachenmayer M. L. Wang J. He L. Poulose S. M. Komatsu M.. (2012). Disrupted autophagy leads to dopaminergic axon and dendrite degeneration and promotes presynaptic accumulation of α-synuclein and LRRK2 in the brain. J. Neurosci. 32, 7585–7593. 10.1523/JNEUROSCI.5809-11.2012 22649237 PMC3382107

[B84] Frost B. Hemberg M. Lewis J. Feany M. B. (2014). Tau promotes neurodegeneration through global chromatin relaxation. Nat. Neurosci. 17, 357–366. 10.1038/nn.3639 24464041 PMC4012297

[B85] Fu K.-Y. Dai L.-G. Chiu I.-M. Chen J.-R. Hsu S.-H. (2011). Sciatic nerve regeneration by microporous nerve conduits seeded with glial cell line-derived neurotrophic factor or brain-derived neurotrophic factor gene transfected neural stem cells. Artif. Organs 35, 363–372. 10.1111/j.1525-1594.2010.01105.x 21314831

[B86] Fulda S. Gorman A. M. Hori O. Samali A. (2010). Cellular stress responses: cell survival and cell death. Int. J. Cell Biol. 2010, 1–23. 10.1155/2010/214074 20182529 PMC2825543

[B87] Furukawa K. Barger S. W. Blalock E. M. Mattson M. P. (1996). Activation of K channels and suppression of neuronal activity by secreted β-amyloid-precursor protein. Nature 379, 74–78. 10.1038/379074a0 8538744

[B88] Galluzzi L. Pietrocola F. Levine B. Kroemer G. (2014). Metabolic control of autophagy. Cell 159, 1263–1276. 10.1016/j.cell.2014.11.006 25480292 PMC4500936

[B89] Gao H. M. Kotzbauer P. T. Uryu K. Leight S. Trojanowski J. Q. Lee V. M. Y. (2008). Neuroinflammation and oxidation/nitration of alpha-synuclein linked to dopaminergic neurodegeneration. J. Neurosci. 28, 7687–7698. 10.1523/JNEUROSCI.0143-07.2008 18650345 PMC2702093

[B90] Gatto R. G. Chu Y. Ye A. Q. Price S. D. Tavassoli E. Buenaventura A.. (2015). Analysis of YFP(J16)-R6/2 reporter mice and postmortem brains reveals early pathology and increased vulnerability of callosal axons in Huntington's disease. Hum. Mol. Genet. 24, 5285–5298. 10.1093/hmg/ddv248 26123489 PMC4550824

[B91] Genzer Y. Dadon M. Burg C. Chapnik N. Froy O. (2016). Effect of dietary fat and the circadian clock on the expression of brain-derived neurotrophic factor (BDNF). Mol. Cell. Endocrinol. 430, 49–55. 10.1016/j.mce.2016.04.015 27113028

[B92] Gerdts J. Brace E. J. Sasaki Y. DiAntonio A. Milbrandt J. (2015). SARM1 activation triggers axon degeneration locally via NAD^+^ destruction. Science 348, 453–457. 10.1126/science.1258366 25908823 PMC4513950

[B93] Gerhard A. Pavese N. Hotton G. Turkheimer F. Es M. Hammers A. (2006). *In vivo* imaging of microglial activation with [11C](R)-PK11195 PET in idiopathic Parkinson's disease. Neurobiol. Dis. 21, 404–412. 10.1016/j.nbd.2005.08.002 16182554

[B94] Ghosh D. LeVault K. R. Barnett A. J. Brewer G. J. (2012). A reversible early oxidized redox state that precedes macromolecular ros damage in aging nontransgenic and 3xTg-AD mouse neurons. J. Neurosci. 32, 5821–5832. 10.1523/JNEUROSCI.6192-11.2012 22539844 PMC3376759

[B95] Gillen C. Jander S. Stoll G. (1998). Sequential expression of mRNA for proinflammatory cytokines and interleukin-10 in the rat peripheral nervous system: comparison between immune-mediated demyelination and wallerian degeneration. J. Neurosci. Res. 51, 489–496. 10.1002/(SICI)1097-4547(19980215)51:4<489::AID-JNR8>3.0.CO;2-8 9514202

[B96] Gomez-Sanchez J. A. Gomis-Coloma C. Morenilla-Palao C. Peiro G. Serra E. Serrano M.. (2013). Epigenetic induction of the Ink4a/Arf locus prevents Schwann cell overproliferation during nerve regeneration and after tumorigenic challenge. Brain 136, 2262–2278. 10.1093/brain/awt130 23748155

[B97] Gonzalez-Horta A. (2015). The interaction of alpha-synuclein with membranes and its implication in Parkinson's Disease: a literature review. Nat. Prod. Commun. 10, 1775–1778. 26669123

[B98] Gowers I. R. Walters K. Kiss-Toth E. Read R. C. Duff G. W. Wilson A. G. (2011). Age-related loss of CpG methylation in the tumour necrosis factor promoter. Cytokine 56, 792–797. 10.1016/j.cyto.2011.09.009 22004920

[B99] Grayson M. (2010). Nutrigenomics. Nature 468:S1. 10.1038/468S1a 21179075

[B100] Hamilton R. Walsh M. Singh R. Rodriguez K. Gao X. Rahman M. M.. (2016). Oxidative damage to myelin proteins accompanies peripheral nerve motor dysfunction in aging C57BL/6 male mice. J. Neurol. Sci. 370, 47–52. 10.1016/j.jns.2016.09.021 27772785

[B101] Hampton D. W. Webber D. J. Bilican B. Goedert M. Spillantini M. G. Chandran S. (2010). Cell-mediated neuroprotection in a mouse model of human tauopathy. J. Neurosc. 30, 9973–9983. 10.1523/JNEUROSCI.0834-10.2010 20668182 PMC6633376

[B102] Han B. H. Zhou M.-L. Johnson A. W. Singh I. Liao F. Vellimana A. K.. (2015). Contribution of reactive oxygen species to cerebral amyloid angiopathy, vasomotor dysfunction, and microhemorrhage in aged Tg2576 mice. Proc. Natl. Acad. Sci. U.S.A. 112, E881–E890. 10.1073/pnas.1414930112 25675483 PMC4345564

[B103] Han S. Choi J.-R. Soon Shin K. Kang S. J. (2012). Resveratrol upregulated heat shock proteins and extended the survival of G93A-SOD1 mice. Brain Res. 1483, 112–117. 10.1016/j.brainres.2012.09.022 23000195

[B104] Harding H. P. Zhang Y. Ron D. (1999). Protein translation and folding are coupled by an endoplasmic-reticulum-resident kinase. Nature 397, 271–274. 10.1038/16729 9930704

[B105] Harman D. (1956). Aging: a theory based on free radical and radiation chemistry. J. Gerontol. 11, 298–300. 10.1093/geronj/11.3.298 13332224

[B106] Haze K. Yoshida H. Yanagi H. Yura T. Mori K. (1999). Mammalian transcription factor ATF6 is synthesized as a transmembrane protein and activated by proteolysis in response to endoplasmic reticulum stress. Mol. Biol. Cell 10, 3787–3799. 10.1091/mbc.10.11.3787 10564271 PMC25679

[B107] He M. Ding Y. Chu C. Tang J. Xiao Q. Luo Z.-G. (2016). Autophagy induction stabilizes microtubules and promotes axon regeneration after spinal cord injury. Proc. Natl. Acad. Sci. U.S.A. 113, 11324–11329. 10.1073/pnas.1611282113 27638205 PMC5056063

[B108] Henis-Korenblit S. Zhang P. Hansen M. McCormick M. Lee S.-J. Cary M.. (2010). Insulin/IGF-1 signaling mutants reprogram ER stress response regulators to promote longevity. Proc. Natl. Acad. Sci. U.S.A. 107, 9730–9735. 10.1073/pnas.1002575107 20460307 PMC2906894

[B109] Hensley K. Carney J. M. Mattson M. P. Aksenova M. Harris M. Wu J. F.. (1994). A model for beta-amyloid aggregation and neurotoxicity based on free radical generation by the peptide: relevance to Alzheimer disease. Proc. Natl. Acad. Sci. U.S.A. 91, 3270–3274. 10.1073/pnas.91.8.3270 8159737 PMC43558

[B110] Hernandez D. G. Nalls M. A. Gibbs J. R. Arepalli S. van der Brug M. Chong S.. (2011). Distinct DNA methylation changes highly correlated with chronological age in the human brain. Hum. Mol. Genet. 20, 1164–1172. 10.1093/hmg/ddq561 21216877 PMC3043665

[B111] Hetz C. Thielen P. Matus S. Nassif M. Court F. Kiffin R.. (2009). XBP-1 deficiency in the nervous system protects against amyotrophic lateral sclerosis by increasing autophagy. Genes Dev. 23, 2294–2306. 10.1101/gad.1830709 19762508 PMC2758741

[B112] Hill S. J. Mordes D. A. Cameron L. A. Neuberg D. S. Landini S. Eggan K.. (2016). Two familial ALS proteins function in prevention/repair of transcription-associated DNA damage. Proc. Natl. Acad. Sci. U.S.A. 113, E7701–E7709. 10.1073/pnas.1611673113 27849576 PMC5137757

[B113] Höglinger G. U. Rizk P. Muriel M. P. Duyckaerts C. Oertel W. H. Caille I.. (2004). Dopamine depletion impairs precursor cell proliferation in Parkinson disease. Nat. Neurosci. 7, 726–735. 10.1038/nn1265 15195095

[B114] Holth J. K. Patel T. K. Holtzman D. M. (2017). Sleep in Alzheimer's Disease–beyond amyloid. Neurobiol. Sleep Circadian Rhythms 2, 4–14. 10.1016/j.nbscr.2016.08.002 28217760 PMC5312809

[B115] Hoozemans J. J. M. van Haastert E. S. Nijholt D. A. T. Rozemuller A. J. M. Eikelenboom P. Scheper W. (2009). The unfolded protein response is activated in pretangle neurons in Alzheimer's disease hippocampus. Am. J. Pathol. 174, 1241–1251. 10.2353/ajpath.2009.080814 19264902 PMC2671357

[B116] Hoozemans J. J. M. Veerhuis R. Van Haastert E. S. Rozemuller J. M. Baas F. Eikelenboom P.. (2005). The unfolded protein response is activated in Alzheimer's disease. Acta Neuropathol. 110, 165–172. 10.1007/s00401-005-1038-0 15973543

[B117] Hu Y. Park K. K. Yang L. Wei X. Yang Q. Cho K.-S.. (2012). Differential effects of unfolded protein response pathways on axon injury-induced death of retinal ganglion cells. Neuron 73, 445–452. 10.1016/j.neuron.2011.11.026 22325198 PMC3278720

[B118] Hussain S. G. Ramaiah K. V. A. (2007). Reduced eIF2α phosphorylation and increased proapoptotic proteins in aging. Biochem. Biophys. Res. Commun. 355, 365–370. 10.1016/j.bbrc.2007.01.156 17300747

[B119] Imai Y. Soda M. Inoue H. Hattori N. Mizuno Y. Takahashi R. (2001). An unfolded putative transmembrane polypeptide, which can lead to endoplasmic reticulum stress, is a substrate of Parkin. Cell 105, 891–902. 10.1016/S0092-8674(01)00407-X 11439185

[B120] Imaizumi Y. Okada Y. Akamatsu W. Koike M. Kuzumaki N. Hayakawa H.. (2012). Mitochondrial dysfunction associated with increased oxidative stress and alpha-synuclein accumulation in PARK2 iPSC-derived neurons and postmortem brain tissue. Mol. Brain 5:35. 10.1186/1756-6606-5-35 23039195 PMC3546866

[B121] Inoue K. Tsutsui H. Akatsu H. Hashizume Y. Matsukawa N. Yamamoto T.. (2013). Metabolic profiling of Alzheimer's disease brains. Sci. Rep. 3, 795–799. 10.1038/srep02364 23917584 PMC3734482

[B122] Ito Y. Ofengeim D. Najafov A. Das S. Saberi S. Li Y.. (2016). RIPK1 mediates axonal degeneration by promoting inflammation and necroptosis in ALS. Science 353, 603–608. 10.1126/science.aaf6803 27493188 PMC5444917

[B123] Jasper H. (2013). Sirtuins: longevity focuses on NAD. Nat. Chem. Biol. 9, 666–667. 10.1038/nchembio.1369 24141218

[B124] Jeong M.-A. Plunet W. Streijger F. Lee J. H. T. Plemel J. R. Park S.. (2011). Intermittent fasting improves functional recovery after rat thoracic contusion spinal cord injury. J. Neurotrauma 28, 479–492. 10.1089/neu.2010.1609 21219083 PMC3119327

[B125] Johnson R. Zuccato C. Belyaev N. D. Guest D. J. Cattaneo E. Buckley N. J. (2008). A microRNA-based gene dysregulation pathway in Huntington's disease. Neurobiol. Dis. 29, 438–445. 10.1016/j.nbd.2007.11.001 18082412

[B126] Johnson V. E. Stewart W. Smith D. H. (2011). Widespread tau and amyloid-beta pathology many years after a single traumatic brain injury in humans. Brain Pathol. 22, 142–149. 10.1111/j.1750-3639.2011.00513.x 21714827 PMC3979351

[B127] Jonsson T. Stefansson H. Steinberg S. Jonsdottir I. Jonsson P. V. Snaedal J.. (2013). Variant of TREM2 associated with the risk of Alzheimer's disease. N. Engl. J. Med. 368, 107–116. 10.1056/NEJMoa1211103 23150908 PMC3677583

[B128] Jowaed A. Schmitt I. Kaut O. Wullner U. (2010). Methylation regulates alpha-synuclein expression and is decreased in parkinson“s disease patients” brains. J. Neurosci. 30, 6355–6359. 10.1523/JNEUROSCI.6119-09.2010 20445061 PMC6632710

[B129] Julien J.-P. Kriz J. (2006). Transgenic mouse models of amyotrophic lateral sclerosis. Biochim. Biophys. Acta Mol. Basis Dis. 1762, 1013–1024. 10.1016/j.bbadis.2006.03.006 16675207

[B130] Karin M. Clevers H. (2016). Reparative inflammation takes charge of tissue regeneration. Nature 529, 307–315. 10.1038/nature17039 26791721 PMC5228603

[B131] Kashiwaya Y. Bergman C. Lee J.-H. Wan R. King M. T. Mughal M. R.. (2013). A ketone ester diet exhibits anxiolytic and cognition-sparing properties, and lessens amyloid and tau pathologies in a mouse model of Alzheimer's disease. Neurobiol. Aging 34, 1530–1539. 10.1016/j.neurobiolaging.2012.11.023 23276384 PMC3619192

[B132] Kashiwaya Y. Takeshima T. Mori N. Nakashima K. Clarke K. Veech R. L. (2000). D-beta-hydroxybutyrate protects neurons in models of Alzheimer's and Parkinson's disease. Proc. Natl. Acad. Sci. U.S.A. 97, 5440–5444. 10.1073/pnas.97.10.5440 10805800 PMC25847

[B133] Keller L. Lincoln B. Albasi S. Frendo N. Freund R. Keller L. (2015). Drosophila neuronal injury follows a temporal sequence of cellular events leading to degeneration at the neuromuscular junction. J. Exp. Neurosci. 9(Suppl. 2), 1–9. 10.4137/JEN.S25516 26512206 PMC4612769

[B134] Kennedy B. K. Berger S. L. Brunet A. Campisi J. Cuervo A. M. Epel E. S.. (2014). Geroscience: linking aging to chronic disease. Cell 159, 709–713. 10.1016/j.cell.2014.10.039 25417146 PMC4852871

[B135] Kerr J. S. Adriaanse B. A. Greig N. H. Mattson M. P. Cader M. Z. Bohr V. A.. (2017). Mitophagy and Alzheimer's Disease: cellular and molecular mechanisms. Trends Neurosci. 40, 151–166. 10.1016/j.tins.2017.01.002 28190529 PMC5341618

[B136] Kigerl K. A. Gensel J. C. Ankeny D. P. Alexander J. K. Donnelly D. J. Popovich P. G. (2009). Identification of two distinct macrophage subsets with divergent effects causing either neurotoxicity or regeneration in the injured mouse spinal cord. J. Neurosci. 29, 13435–13444. 10.1523/JNEUROSCI.3257-09.2009 19864556 PMC2788152

[B137] Kikuchi H. Furuta A. Nishioka K.-I. Suzuki S. Nakabeppu Y. Iwaki T. (2002). Impairment of mitochondrial DNA repair enzymes against accumulation of 8-oxo-guanine in the spinal motor neurons of amyotrophic lateral sclerosis. Acta Neuropathol. 103, 408–414. 10.1007/s00401-001-0480-x 11904761

[B138] Kim H. J. Jung K. J. Yu B. P. Cho C. G. Choi J. S. Chung H. Y. (2002). Modulation of redox-sensitive transcription factors by calorie restriction during aging. Mech. Ageing Dev. 123, 1589–1595. 10.1016/S0047-6374(02)00094-5 12470896

[B139] Kim S. R. Kareva T. Yarygina O. Kholodilov N. Burke R. E. (2009). AAV transduction of dopamine neurons with constitutively active Rheb protects from neurodegeneration and mediates axon regrowth. Mol. Ther. 20, 275–286. 10.1038/mt.2011.213 22008911 PMC3277224

[B140] Kim Y. Zhou P. Qian L. Chuang J.-Z. Lee J. Li C.. (2007). MyD88-5 links mitochondria, microtubules, and JNK3 in neurons and regulates neuronal survival. J. Exp. Med. 204, 2063–2074. 10.1084/jem.20070868 17724133 PMC2118693

[B141] Knowles R. B. Wyart C. Buldyrev S. V. Cruz L. Urbanc B. Hasselmo M. E.. (1999). Plaque-induced neurite abnormalities: implications for disruption of neural networks in Alzheimer's disease. Proc. Natl. Acad. Sci. U.S.A. 96, 5274–5279. 10.1073/pnas.96.9.5274 10220456 PMC21854

[B142] Koh K. Evans J. M. Hendricks J. C. Sehgal A. (2006). A Drosophila model for age-associated changes in sleep:wake cycles. Proc. Natl. Acad. Sci. U.S.A. 103, 13843–13847. 10.1073/pnas.0605903103 16938867 PMC1564207

[B143] Komatsu M. Wang Q. J. Holstein G. R. Victor L Friedrich J. Iwata J.-I. Kominami E.. (2007). Essential role for autophagy protein Atg7 in the maintenance of axonal homeostasis and the prevention of axonal degeneration. Proc. Natl. Acad. Sci. U.S.A. 104, 14489–14494. 10.1073/pnas.0701311104 17726112 PMC1964831

[B144] Körner S. Böselt S. Thau N. Rath K. J. Dengler R. Petri S. (2013). Differential sirtuin expression patterns in amyotrophic lateral sclerosis (ALS) postmortem tissue: neuroprotective or neurotoxic properties of sirtuins in ALS? Neurodegener. Dis. 11, 141–152. 10.1159/000338048 22796962

[B145] Kruegel U. Robison B. Dange T. Kahlert G. Delaney J. R. Kotireddy S.. (2011). Elevated proteasome capacity extends replicative lifespan in *Saccharomyces cerevisiae*. PLoS Genet. 7:e1002253. 10.1371/journal.pgen.1002253 21931558 PMC3169524

[B146] Kurowska Z. Kordower J. H. Stoessl A. J. Burke R. E. Brundin P. Yue Z.. (2016). Is axonal degeneration a key early event in Parkinson's Disease? J. Parkinsons Dis. 6, 703–707. 10.3233/JPD-160881 27497486

[B147] Labbadia J. Morimoto R. I. (2015). The biology of proteostasis in aging and disease. Annu. Rev. Biochem. 84, 435–464. 10.1146/annurev-biochem-060614-033955 25784053 PMC4539002

[B148] Labunskyy V. M. Gerashchenko M. V. Delaney J. R. Kaya A. Kennedy B. K. Kaeberlein M.. (2014). Lifespan extension conferred by endoplasmic reticulum secretory pathway deficiency requires induction of the unfolded protein response. PLoS Genet. 10:e1004019. 10.1371/journal.pgen.1004019 24391512 PMC3879150

[B149] Ladd A. C. Brohawn D. G. Thomas R. R. Keeney P. M. Berr S. S. Khan S. M.. (2017). RNA-seq analyses reveal that cervical spinal cords and anterior motor neurons from amyotrophic lateral sclerosis subjects show reduced expression of mitochondrial DNA-encoded respiratory genes, and rhTFAM may correct this respiratory deficiency. Brain Res. 1667, 74–83. 10.1016/j.brainres.2017.05.010 28511992

[B150] Lam L. Chin L. Halder R. C. Sagong B. Famenini S. Sayre J.. (2016). Epigenetic changes in T-cell and monocyte signatures and production of neurotoxic cytokines in ALS patients. FASEB J. 30, 3461–3473. 10.1096/fj.201600259RR 27368295 PMC5024692

[B151] Launay N. Aguado C. Fourcade S. Ruiz M. Grau L. Riera J.. (2014). Autophagy induction halts axonal degeneration in a mouse model of X-adrenoleukodystrophy. Acta Neuropathol. 129, 399–415. 10.1007/s00401-014-1378-8 25549970 PMC4331612

[B152] Lee S.-T. Chu K. Im W.-S. Yoon H.-J. Im J.-Y. Park J.-E.. (2011). Altered microRNA regulation in Huntington's disease models. Exp. Neurol. 227, 172–179. 10.1016/j.expneurol.2010.10.012 21035445

[B153] Li H. Li S. H. Yu Z. X. Shelbourne P. Li X. J. (2001). Huntingtin aggregate-associated axonal degeneration is an early pathological event in Huntington's disease mice. J. Neurosci. 21, 8473–8481. 11606636 10.1523/JNEUROSCI.21-21-08473.2001 PMC6762783

[B154] Li J.-Y. Conforti L. (2013). Axonopathy in Huntington's disease. Exp. Neurol. 246, 62–71. 10.1016/j.expneurol.2012.08.010 22921535

[B155] Li L.-S. Lu Y.-L. Nie J. Xu Y.-Y. Zhang W. Yang W.-J. (2017). Dendrobium nobileLindl alkaloid, a novel autophagy inducer, protects against axonal degeneration induced by Aβ 25-35in hippocampus neurons *in vitro*. CNS Neurosci. Ther. 23, 329–340. 10.1111/cns.12678 28261990 PMC6492701

[B156] Li S. Yang L. Selzer M. E. Hu Y. (2013). Neuronal endoplasmic reticulum stress in axon injury and neurodegeneration. Ann. Neurol. 74, 768–777. 10.1002/ana.24005 23955583 PMC3963272

[B157] Li Y. Liu W. Oo T. F. Wang L. Tang Y. Jackson-Lewis V.. (2009). Mutant LRRK2R1441G BAC transgenic mice recapitulate cardinal features of Parkinson's disease. Nat. Neurosci. 12, 826–828. 10.1038/nn.2349 19503083 PMC2845930

[B158] Liefner M. Siebert H. Sachse T. Michel U. Kollias G. Brück W. (2000). The role of TNF-alpha during Wallerian degeneration. J. Neuroimmunol. 108, 147–152. 10.1016/S0165-5728(00)00262-9 10900348

[B159] Lin C.-W. Liu H.-Y. Chen C.-Y. Hsueh Y.-P. (2014). Neuronally-expressed Sarm1 regulates expression of inflammatory and antiviral cytokines in brains. Innate Immun. 20, 161–172. 10.1177/1753425913485877 23751821

[B160] Lindvall O. (2013). Developing dopaminergic cell therapy for Parkinson's disease–give up or move forward? Mov. Disord. 28, 268–273. 10.1002/mds.25378 23401015

[B161] Lipinski M. M. Zheng B. Lu T. Yan Z. Py B. F. Ng A.. (2010). Genome-wide analysis reveals mechanisms modulating autophagy in normal brain aging and in Alzheimer's disease. Proc. Natl. Acad. Sci. U.S.A. 107, 14164–14169. 10.1073/pnas.1009485107 20660724 PMC2922576

[B162] Lipsky M. S. King M. (2015). Biological theories of aging. Dis. Mon. 61, 460–466. 10.1016/j.disamonth.2015.09.005 26490576

[B163] Liu H. Wang H. Shenvi S. Hagen T. M. Liu R. M. (2004). Glutathione metabolism during aging and in Alzheimer Disease. Ann. N. Y. Acad. Sci. 1019, 346–349. 10.1196/annals.1297.059 15247041

[B164] Liu K. Lu Y. Lee J. K. Samara R. Willenberg R. Sears-Kraxberger I.. (2010). PTEN deletion enhances the regenerative ability of adult corticospinal neurons. Nat. Neurosci. 13, 1075–1081. 10.1038/nn.2603 20694004 PMC2928871

[B165] Liu L. Zhang K. Sandoval H. Yamamoto S. Jaiswal M. Sanz E.. (2015). Glial lipid droplets and ROS induced by mitochondrial defects promote neurodegeneration. Cell 160, 177–190. 10.1016/j.cell.2014.12.019 25594180 PMC4377295

[B166] Logroscino G. Marder K. Graziano J. Freyer G. Slavkovich V. LoIacono N.. (1997). Altered systemic iron metabolism in Parkinson's disease. Neurology 49, 714–717. 10.1212/WNL.49.3.714 9305329

[B167] Lopez G. Bayulkem K. Hallett M. (2016). Progressive supranuclear palsy (PSP): Richardson syndrome and other PSP variants. Acta Neurol. Scand. 134, 242–249. 10.1111/ane.12546 27070344 PMC7292631

[B168] Lopez-Gonzalez R. Lu Y. Gendron T. F. Karydas A. Tran H. Yang D.. (2016). Poly(GR) in C9ORF72-related ALS/FTD compromises mitochondrial function and increases oxidative stress and DNA damage in iPSC-derived motor neurons. Neuron 92, 383–391. 10.1016/j.neuron.2016.09.015 27720481 PMC5111366

[B169] Lucchinetti C. Bruck W. (2004). The pathology of primary progressive multiple sclerosis. Mult. Scler. 10(Suppl. 1), S23–S30. 10.1191/1352458504ms1027oa 15218806

[B170] Ludolph A. C. Bendotti C. Blaugrund E. Hengerer B. Löffler J. P. Martin J.. (2009). Guidelines for the preclinical *in vivo* evaluation of pharmacological active drugs for ALS/MND: report on the 142nd ENMC international workshop. Amyotroph. Lateral Scler. 8, 217–223. 10.1080/17482960701292837 17653919

[B171] Mack T. G. A. Reiner M. Beirowski B. Mi W. Emanuelli M. Wagner D.. (2001). Wallerian degeneration of injured axons and synapses is delayed by a Ube4b/Nmnat chimeric gene. Nat. Neurosci. 4, 1199–1206. 10.1038/nn770 11770485

[B172] MacPherson K. P. Sompol P. Kannarkat G. T. Chang J. Sniffen L. Wildner M. E. (2017). Peripheral administration of the soluble TNF inhibitor XPro1595 modifies brain immune cell profiles, decreases beta-amyloid plaque load, and rescues impaired long-term potentiation in 5xFAD mice. Neurobiol. Dis. 10, 81–95. 10.1016/j.nbd.2017.02.010 PMC5464789 28237313

[B173] Maday S. Holzbaur E. L. F. (2014). Autophagosome biogenesis in primary neurons follows an ordered and spatially regulated pathway. Dev. Cell 30, 71–85. 10.1016/j.devcel.2014.06.001 25026034 PMC4109719

[B174] Madeo F. Zimmermann A. Maiuri M. C. Kroemer G. (2015). Essential role for autophagy in life span extension. J. Clin. Invest. 125, 85–93. 10.1172/JCI73946 25654554 PMC4382258

[B175] Madji Hounoum B. Mavel S. Coque E. Patin F. Vourc'h P. Marouillat S.. (2017). Wildtype motoneurons, ALS-Linked SOD1 mutation and glutamate profoundly modify astrocyte metabolism and lactate shuttling. Glia 65, 592–605. 10.1002/glia.23114 28139855

[B176] Madorsky I. Opalach K. Waber A. Verrier J. D. Solmo C. Foster T.. (2009). Intermittent fasting alleviates the neuropathic phenotype in a mouse model of Charcot-Marie-Tooth disease. Neurobiol. Dis. 34, 146–154. 10.1016/j.nbd.2009.01.002 19320048 PMC2757933

[B177] Maglemose R. Hedegaard A. Lehnhoff J. Dimintiyanova K. P. Moldovan M. Grøndahl L.. (2017). Potassium channel abnormalities are consistent with early axon degeneration of motor axons in the G127X SOD1 mouse model of amyotrophic lateral sclerosis. Exp. Neurol. 292, 154–167. 10.1016/j.expneurol.2017.03.008 28322742

[B178] Magrané J. Cortez C. Gan W.-B. Manfredi G. (2014). Abnormal mitochondrial transport and morphology are common pathological denominators in SOD1 and TDP43 ALS mouse models. Hum. Mol. Genet. 23, 1413–1424. 10.1093/hmg/ddt528 24154542 PMC3929084

[B179] Majmundar A. J. Wong W. J. Simon M. C. (2010). Hypoxia-inducible factors and the response to hypoxic stress. Mol. Cell 40, 294–309. 10.1016/j.molcel.2010.09.022 20965423 PMC3143508

[B180] Malapati H. Millen S. M. and J. Buchser W. (2017). The axon degeneration gene SARM1 is evolutionarily distinct from other TIR domain-containing proteins. Mol. Genet. Genomics 292, 909–922. 10.1007/s00438-017-1320-6 28447196

[B181] Mantuano E. Henry K. Yamauchi T. Hiramatsu N. Yamauchi K. Orita S.. (2011). The unfolded protein response is a major mechanism by which LRP1 regulates schwann cell survival after injury. J. Neurosci. 31, 13376–13385. 10.1523/JNEUROSCI.2850-11.2011 21940431 PMC3188465

[B182] Marangoni M. Adalbert R. Janeckova L. Patrick J. Kohli J. Coleman M. P.. (2014). Age-related axonal swellings precede other neuropathological hallmarks in a knock-in mouse model of Huntington's disease. Neurobiol. Aging 35, 2382–2393. 10.1016/j.neurobiolaging.2014.04.024 24906892

[B183] Marsh S. E. Blurton-Jones M. (2017). Neural stem cell therapy for neurodegenerative disorders: the role of neurotrophic support. Neurochem. Int. 106, 94–100. 10.1016/j.neuint.2017.02.006 28219641 PMC5446923

[B184] Martin L. J. Gertz B. Pan Y. Price A. C. Molkentin J. D. Chang Q. (2009). The mitochondrial permeability transition pore in motor neurons: involvement in the pathobiology of ALS mice. Exp. Neurol. 218, 333–346. 10.1016/j.expneurol.2009.02.015 19272377 PMC2710399

[B185] Martin L. J. Semenkow S. Hanaford A. Wong M. (2014). The mitochondrial permeability transition pore regulates Parkinson's disease development in mutant α-synuclein transgenic mice. Neurobiol. Aging 35, 1132–1152. 10.1016/j.neurobiolaging.2013.11.008 24325796 PMC3948207

[B186] Martin S. M. O'Brien G. S. Portera-Cailliau C. Sagasti A. (2010). Wallerian degeneration of zebrafish trigeminal axons in the skin is required for regeneration and developmental pruning. Development 137, 3985–3994. 10.1242/dev.053611 21041367 PMC2976282

[B187] Martínez G. Duran-Aniotz C. Cabral-Miranda F. Vivar J. P. Hetz C. (2017). Endoplasmic reticulum proteostasis impairment in aging. Aging Cell 9, e1003433–e1003439. 10.1111/acel.12599 PMC5506418 28436203

[B188] Maswood N. Young J. Tilmont E. Zhang Z. Gash D. M. Gerhardt G. A.. (2004). Caloric restriction increases neurotrophic factor levels and attenuates neurochemical and behavioral deficits in a primate model of Parkinson's disease. Proc. Natl. Acad. Sci. U.S.A. 101, 18171–18176. 10.1073/pnas.0405831102 15604149 PMC539733

[B189] Mattison J. A. Colman R. J. Beasley T. M. Allison D. B. Kemnitz J. W. Roth G. S.. (2017). Caloric restriction improves health and survival of rhesus monkeys. Nat. Commun. 8, 1–12. 10.1038/ncomms14063 28094793 PMC5247583

[B190] Mattson M. P. (2008). Hormesis defined. Ageing Res. Rev. 7, 1–7. 10.1016/j.arr.2007.08.007 18162444 PMC2248601

[B191] Mattson M. P. Cheng B. Davis D. Bryant K. Lieberburg I. Rydel R. E. (1992). beta-Amyloid peptides destabilize calcium homeostasis and render human cortical neurons vulnerable to excitotoxicity. J. Neurosci. 12, 376–389. 1346802 10.1523/JNEUROSCI.12-02-00376.1992 PMC6575616

[B192] Maze I. Shen L. Zhang B. Garcia B. A. Shao N. Mitchell A.. (2014). Analytical tools and current challenges in the modern era of neuroepigenomics. Nat. Neurosci. 17, 1476–1490. 10.1038/nn.3816 25349914 PMC4262187

[B193] Means J. C. Venkatesan A. Gerdes B. Fan J.-Y. Bjes E. S. Price J. L. (2015). Drosophila spaghetti and doubletime link the circadian clock and light to caspases, apoptosis and tauopathy. PLoS Genet. 11, e1005171–e1005124. 10.1371/journal.pgen.1005171 25951229 PMC4423883

[B194] Mendioroz M. Celarain N. Altuna M. de Gordoa J. S.-R. Zelaya M. V. Roldán M.. (2016). CRTC1 gene is differentially methylated in the human hippocampus in Alzheimer's disease. Alzheimers Res. Ther. 8:15. 10.1186/s13195-016-0183-0 27094739 PMC4837517

[B195] Meyer-Luehmann M. Spires-Jones T. L. Prada C. Garcia-Alloza M. de Calignon A. Rozkalne A.. (2008). Rapid appearance and local toxicity of amyloid-β plaques in a mouse model of Alzheimer's disease. Nature 451, 720–724. 10.1038/nature06616 18256671 PMC3264491

[B196] Migliore L. Fontana I. Trippi F. Colognato R. Coppedè F. Tognoni G.. (2005). Oxidative DNA damage in peripheral leukocytes of mild cognitive impairment and AD patients. Neurobiol. Aging 26, 567–573. 10.1016/j.neurobiolaging.2004.07.016 15708428

[B197] Miller K. N. Burhans M. S. Clark J. P. Howell P. R. Polewski M. A. DeMuth T. M.. (2017). Aging and caloric restriction impact adipose tissue, adiponectin, and circulating lipids. Aging Cell 16, 497–507. 10.1111/acel.12575 28156058 PMC5418198

[B198] Mink M. Csiszar K. (2005). *SARM1:* a candidate gene in the onset of hereditary infectious/inflammatory diseases. Clin. Immunol. 115, 333–334. 10.1016/j.clim.2005.03.002 15893701

[B199] Mogi M. Harada M. Riederer P. Narabayashi H. Fujita K. Nagatsu T. (1994). Tumor necrosis factor-α (TNF-α) increases both in the brain and in the cerebrospinal fluid from parkinsonian patients. Neurosci. Lett. 165, 208–210. 10.1016/0304-3940(94)90746-3 8015728

[B200] Moldovan M. Alvarez S. Krarup C. (2009). Motor axon excitability during Wallerian degeneration. Brain 132, 511–523. 10.1093/brain/awn332 19074190

[B201] Molofsky A. V. Slutsky S. G. Joseph N. M. He S. Pardal R. Krishnamurthy J.. (2006). Increasing p16INK4a expression decreases forebrain progenitors and neurogenesis during ageing. Nature 443, 448–452. 10.1038/nature05091 16957738 PMC2586960

[B202] Moreno J. A. Halliday M. Molloy C. Radford H. Verity N. Axten J. M.. (2013). Oral treatment targeting the unfolded protein response prevents neurodegeneration and clinical disease in prion-infected mice. Sci. Transl. Med. 5, 206ra138–206ra138. 10.1126/scitranslmed.3006767 24107777

[B203] Moreno J. A. Radford H. Peretti D. Steinert J. R. Verity N. Martin M. G. (2012). Sustained translational repression by eIF2α-P mediates prion neurodegeneration. Nature 354, 707–714. 10.1038/nature11058 PMC3378208 22622579

[B204] Morrow G. (2004). Overexpression of the small mitochondrial Hsp22 extends Drosophila life span and increases resistance to oxidative stress. FASEB J. 18, 598–599. 10.1096/fj.03-0860fje 14734639

[B205] Mucke L. Masliah E. Yu G. Q. Mallory M. Rockenstein E. M. Tatsuno G.. (2000). High-level neuronal expression of abeta 1-42 in wild-type human amyloid protein precursor transgenic mice: synaptotoxicity without plaque formation. J. Neurosci. 20, 4050–4058. 10818140 10.1523/JNEUROSCI.20-11-04050.2000 PMC6772621

[B206] Mukherjee P. Woods T. A. Moore R. A. Peterson K. E. (2013). Activation of the innate signaling molecule MAVS by bunyavirus infection upregulates the adaptor protein SARM1, leading to neuronal death. Immunity 38, 705–716. 10.1016/j.immuni.2013.02.013 23499490 PMC4783152

[B207] Murakami T. Warita H. Hayashi T. Sato K. Manabe Y. Mizuno S.. (2001). A novel SOD1 gene mutation in familial ALS with low penetrance in females. J. Neurol. Sci. 189, 45–47. 10.1016/S0022-510X(01)00558-5 11535232

[B208] Musiek E. S. Holtzman D. M. (2016). Mechanisms linking circadian clocks, sleep, and neurodegeneration. Science 354, 1004–1008. 10.1126/science.aah4968 27885006 PMC5219881

[B209] Mythri R. B. Venkateshappa C. Harish G. Mahadevan A. Muthane U. B. Yasha T. C.. (2011). Evaluation of markers of oxidative stress, antioxidant function and astrocytic proliferation in the striatum and Frontal cortex of Parkinson's Disease brains. Neurochem. Res. 36, 1452–1463. 10.1007/s11064-011-0471-9 21484266

[B210] Naidoo N. Ferber M. Master M. Zhu Y. Pack A. I. (2008). Aging impairs the unfolded protein response to sleep deprivation and leads to proapoptotic signaling. J. Neurosci. 28, 6539–6548. 10.1523/JNEUROSCI.5685-07.2008 18579727 PMC2925257

[B211] Nakahata Y. Sahar S. Astarita G. Kaluzova M. Sassone-Corsi P. (2009). Circadian control of the NAD^+^ salvage pathway by CLOCK-SIRT1. Science 324, 654–657. 10.1126/science.1170803 19286518 PMC6501775

[B212] Nelson P. T. Alafuzoff I. Bigio E. H. Bouras C. Braak H. Cairns N. J.. (2012). Correlation of Alzheimer Disease neuropathologic changes with cognitive status: a review of the literature. J. Neuropathol. Exp. Neurol. 71, 362–381. 10.1097/NEN.0b013e31825018f7 22487856 PMC3560290

[B213] Neukomm L. J. Freeman M. R. (2014). Diverse cellular and molecular modes of axon degeneration. Trends Cell Biol. 24, 515–523. 10.1016/j.tcb.2014.04.003 24780172 PMC4149811

[B214] Ng C. W. Yildirim F. Yap Y. S. Dalin S. Matthews B. J. Velez P. J.. (2013). Extensive changes in DNA methylation are associated with expression of mutant huntingtin. Proc. Natl. Acad. Sci. U.S.A. 110, 2354–2359. 10.1073/pnas.1221292110 23341638 PMC3568325

[B215] Nisoli E. Tonello C. Cardile A. Cozzi V. Bracale R. Tedesco L.. (2005). Calorie restriction promotes mitochondrial biogenesis by inducing the expression of eNOS. Science 310, 314–317. 10.1126/science.1117728 16224023

[B216] Oñate M. Catenaccio A. Martínez G. Armentano D. Parsons G. Kerr B. (2016). Activation of the unfolded protein response promotes axonal regeneration after peripheral nerve injury. *Sci*. Rep. 6:21709 10.1038/srep21709 PMC4764858 26906090

[B217] Orimo S. Amino T. Itoh Y. Takahashi A. Kojo T. Uchihara T.. (2005). Cardiac sympathetic denervation precedes neuronal loss in the sympathetic ganglia in Lewy body disease. Acta Neuropathol. 109, 583–588. 10.1007/s00401-005-0995-7 15933869

[B218] Orimo S. Uchihara T. Nakamura A. Mori F. Kakita A. Wakabayashi K.. (2008). Axonal α-synuclein aggregates herald centripetal degeneration of cardiac sympathetic nerve in Parkinson's disease. Brain 131, 642–650. 10.1093/brain/awm302 18079166

[B219] Osterloh J. M. Yang J. Rooney T. M. Fox A. N. Adalbert R. Powell E. H.. (2012). dSarm/Sarm1 is required for activation of an injury-induced axon death pathway. Science 337, 481–484. 10.1126/science.1223899 22678360 PMC5225956

[B220] Owusu-Ansah E. Song W. Perrimon N. (2013). Muscle mitohormesis promotes longevity via systemic repression of insulin signaling. Cell 155, 699–712. 10.1016/j.cell.2013.09.021 24243023 PMC3856681

[B221] Panneerselvam P. Singh L. P. Ho B. Chen J. Ding J. L. (2012). Targeting of pro-apoptotic TLR adaptor SARM to mitochondria: definition of the critical region and residues in the signal sequence. Biochem. J. 442, 263–271. 10.1042/BJ20111653 22145856

[B222] Park K. K. Liu K. Hu Y. Smith P. D. Wang C. Cai B.. (2008). Promoting axon regeneration in the adult CNS by modulation of the PTEN/mTOR pathway. Science 322, 963–966. 10.1126/science.1161566 18988856 PMC2652400

[B223] Pasinelli P. Brown R. H. (2006). Molecular biology of amyotrophic lateral sclerosis: insights from genetics. Nat. Rev. Neurosci. 7, 710–723. 10.1038/nrn1971 16924260

[B224] Pasinetti G. M. Bilski A. E. Zhao W. (2013). Sirtuins as therapeutic targets of ALS. Cell Res. 23, 1073–1074. 10.1038/cr.2013.94 23856645 PMC3760621

[B225] Patergnani S. Fossati V. Bonora M. Giorgi C. Marchi S. Missiroli S.. (2017). Mitochondria in Multiple Sclerosis: Molecular Mechanisms of Pathogenesis. Int. Rev. Cell Mol. Biol. 328, 49–103. 10.1016/bs.ircmb.2016.08.003 28069137

[B226] Paz Gavilán M. Vela J. Castaño A. Ramos B. del Río J. C. Vitorica J.. (2006). Cellular environment facilitates protein accumulation in aged rat hippocampus. Neurobiol. Aging 27, 973–982. 10.1016/j.neurobiolaging.2005.05.010 15964666

[B227] Penas C. Font-Nieves M. Forés J. Petegnief V. Planas A. Navarro X.. (2011). Autophagy, and BiP level decrease are early key events in retrograde degeneration of motoneurons. Cell Death Differ. 18, 1617–1627. 10.1038/cdd.2011.24 21436843 PMC3172115

[B228] Peng Y. Kim M. J. Hullinger R. O'Riordan K. J. Burger C. Pehar M.. (2016). Improved proteostasis in the secretory pathway rescues Alzheimer's disease in the mouse. Brain 139, 937–952. 10.1093/brain/awv385 26787453 PMC4805081

[B229] Pires A. O. Teixeira F. G. Mendes-Pinheiro B. Serra S. C. Sousa N. Salgado A. J. (2017). Old and new challenges in parkinson's disease therapeutics. Prog. Neurobiol. [Epub ahead of print]. 10.1016/j.pneurobio.2017.04.006 28457671

[B230] Pott Godoy M. C. Tarelli R. Ferrari C. C. Sarchi M. I. Pitossi F. J. (2008). Central and systemic IL-1 exacerbates neurodegeneration and motor symptoms in a model of Parkinson's disease. Brain 131, 1880–1894. 10.1093/brain/awn101 18504291 PMC2442423

[B231] Poulsen E. T. Iannuzzi F. Rasmussen H. F. Maier T. J. Enghild J. J. Jørgensen A. L. (2017). An aberrant phosphorylation of amyloid precursor protein tyrosine regulates its trafficking and the binding to the clathrin endocytic complex in neural stem cells of Alzheimer's Disease Patients. Front. Mol. Neurosci. 10:59 10.3389/fnmol.2017.00059 PMC5350151 28360834

[B232] Prehn K. Jumpertz von Schwartzenberg R. Mai K. Zeitz U. Witte A. V. Hampel D.. (2016). Caloric restriction in older adults—differential effects of weight loss and reduced weight on brain structure and function. Cereb. Cortex 27, 1765–1778. 10.1093/cercor/bhw008 26838769

[B233] Prolo L. M. Takahashi J. S. Herzog E. D. (2005). Circadian rhythm generation and entrainment in astrocytes. J. Neurosci. 25, 404–408. 10.1523/JNEUROSCI.4133-04.2005 15647483 PMC3812245

[B234] Puls I. Jonnakuty C. LaMonte B. H. Holzbaur E. L. F. Tokito M. Mann E.. (2003). Mutant dynactin in motor neuron disease. Nat. Genet. 33, 455–456. 10.1038/ng1123 12627231

[B235] Pyo J.-O. Yoo S.-M. Ahn H.-H. Nah J. Hong S.-H. Kam T.-I.. (2013). Overexpression of Atg5 in mice activates autophagy and extends lifespan. Nat Commun. 4:2300. 10.1038/ncomms3300 23939249 PMC3753544

[B236] Rando T. A. Chang H. Y. (2012). Aging, rejuvenation, and epigenetic reprogramming: resetting the aging clock. Cell 148, 46–57. 10.1016/j.cell.2012.01.003 22265401 PMC3336960

[B237] Ransohoff R. M. (2016). How neuroinflammation contributes to neurodegeneration. Science 353, 777–783. 10.1126/science.aag2590 27540165

[B238] Reed T. T. Pierce W. M. Markesbery W. R. Butterfield D. A. (2009). Proteomic identification of HNE-bound proteins in early Alzheimer disease: insights into the role of lipid peroxidation in the progression of AD. Brain Res. 1274, 66–76. 10.1016/j.brainres.2009.04.009 19374891

[B239] Riar A. K. Burstein S. R. Palomo G. M. Arreguin A. Manfredi G. Germain D. (2017). Sex specific activation of the ERα axis of the mitochondrial UPR (UPRmt) in the G93A-SOD1 mouse model of familial ALS. Hum. Mol. Genet. 26, 1318–1327. 10.1093/hmg/ddx049 28186560 PMC6075578

[B240] Richardson A. G. Schadt E. E. (2014). The role of macromolecular damage in aging and age-related disease. J. Gerontol. Ser. A Biol. Sci. Med. Sci. 69, S28–S32. 10.1093/gerona/glu056 24833583

[B241] Roediger B. Armati P. J. (2003). Oxidative stress induces axonal beading in cultured human brain tissue. Neurobiol. Dis. 13, 222–229. 10.1016/S0969-9961(03)00038-X 12901836

[B242] Rogina B. Helfand S. L. (2004). Sir2 mediates longevity in the fly through a pathway related to calorie restriction. Proc. Natl. Acad. Sci. U.S.A. 101, 15998–16003. 10.1073/pnas.0404184101 15520384 PMC528752

[B243] Rosen D. R. Siddique T. Patterson D. Figlewicz D. A. Sapp P. Hentati A.. (1993). Mutations in Cu/Zn superoxide dismutase gene are associated with familial amyotrophic lateral sclerosis. Nature 362, 59–62. 10.1038/362059a0 8446170

[B244] Rotem N. Magen I. Ionescu A. Gershoni-Emek N. Altman T. Costa C. J.. (2017). ALS Along the Axons - Expression of Coding and Noncoding RNA Differs in Axons of ALS models. Sci. Rep. 7:44500. 10.1038/srep44500 28300211 PMC5353576

[B245] Ruan H. Tang X. D. Chen M.-L. Joiner M.-L. A. Sun G. Brot N.. (2002). High-quality life extension by the enzyme peptide methionine sulfoxide reductase. Proc. Natl. Acad. Sci. U.S.A. 99, 2748–2753. 10.1073/pnas.032671199 11867705 PMC122419

[B246] Rubinsztein D. C. Mariño G. Kroemer G. (2011). Autophagy and aging. Cell 146, 682–695. 10.1016/j.cell.2011.07.030 21884931

[B247] Ruzankina Y. Pinzon-Guzman C. Asare A. Ong T. Pontano L. Cotsarelis G.. (2007). Deletion of the Developmentally essential gene ATR in Adult mice leads to age-related phenotypes and stem cell loss. Cell Stem Cell 1, 113–126. 10.1016/j.stem.2007.03.002 18371340 PMC2920603

[B248] Sadleir K. R. Kandalepas P. C. Buggia-Prévot V. Nicholson D. A. Thinakaran G. Vassar R. (2016). Presynaptic dystrophic neurites surrounding amyloid plaques are sites of microtubule disruption, BACE1 elevation, and increased Aβ generation in Alzheimer's disease. Acta Neuropathologica 132, 235–256. 10.1007/s00401-016-1558-9 26993139 PMC4947125

[B249] Sadri-Vakili G. Bouzou B. Benn C. L. Kim M.-O. Chawla P. Overland R. P.. (2007). Histones associated with downregulated genes are hypo-acetylated in Huntington's disease models. Hum. Mol. Genet. 16, 1293–1306. 10.1093/hmg/ddm078 17409194

[B250] Saha A. R. (2004). Parkinson's disease α-synuclein mutations exhibit defective axonal transport in cultured neurons. J. Cell. Sci. 117, 1017–1024. 10.1242/jcs.00967 14996933

[B251] Saito Y. Hamakubo T. Yoshida Y. Ogawa Y. Hara Y. Fujimura H.. (2009). Preparation and application of monoclonal antibodies against oxidized DJ-1. Significant elevation of oxidized DJ-1 in erythrocytes of early-stage Parkinson disease patients. Neuroscience Lett. 465, 1–5. 10.1016/j.neulet.2009.08.074 19733211

[B252] Salpea P. Russanova V. R. Hirai T. H. Sourlingas T. G. Sekeri-Pataryas K. E. Romero R.. (2012). Postnatal development- and age-related changes in DNA-methylation patterns in the human genome. Nucleic Acids Res. 40, 6477–6494. 10.1093/nar/gks312 22495928 PMC3413121

[B253] Sanchez-Varo R. Trujillo-Estrada L. Sanchez-Mejias E. Torres M. Baglietto-Vargas D. Moreno-Gonzalez I.. (2011). Abnormal accumulation of autophagic vesicles correlates with axonal and synaptic pathology in young Alzheimer's mice hippocampus. Acta Neuropathol. 123, 53–70. 10.1007/s00401-011-0896-x 22020633 PMC3249205

[B254] Sanhueza M. Chai A. Smith C. McCray B. A. Simpson T. I. Taylor J. P.. (2015). Network analyses reveal novel aspects of ALS pathogenesis. PLoS Genet. 11:e1005107. 10.1371/journal.pgen.1005107 25826266 PMC4380362

[B255] Sasaki Y. Nakagawa T. Mao X. DiAntonio A. Milbrandt J. (2016). NMNAT1 inhibits axon degeneration via blockade of SARM1-mediated NAD^+^ depletion. Elife 5:e19749. 10.7554/eLife.19749 27735788 PMC5063586

[B256] Schuldiner O. Yaron A. (2014). Mechanisms of developmental neurite pruning. Cell. Mol. Life Sci. 72, 101–119. 10.1007/s00018-014-1729-6 25213356 PMC5086088

[B257] Schwartzman O. Tanay A. (2015). Single-cell epigenomics: techniques and emerging applications. Nat. Rev. Genet. 16, 716–726. 10.1038/nrg3980 26460349

[B258] Selkoe D. J. Hardy J. (2016). The amyloid hypothesis of Alzheimer's disease at 25 years. EMBO Mol Med. 8, 595–608. 10.15252/emmm.201606210 27025652 PMC4888851

[B259] Sepe S. Milanese C. Gabriels S. Derks K. W. J. Payan-Gomez C. van IJcken W. F. J.. (2016). Inefficient DNA repair is an aging-related modifier of Parkinson's Disease. Cell Rep. 15, 1866–1875. 10.1016/j.celrep.2016.04.071 27210754 PMC4893155

[B260] Serrano-Pozo A. Frosch M. P. Masliah E. Hyman B. T. (2011). Neuropathological ALTERATIONS in Alzheimer Disease. Cold Spring Harb. Perspect. Med. 1, a006189–a006189. 10.1101/cshperspect.a006189 22229116 PMC3234452

[B261] Sharma R. Buras E. Terashima T. Serrano F. Massaad C. A. Hu L.. (2010). Hyperglycemia induces oxidative stress and impairs axonal transport rates in mice. PLoS ONE 5:e13463. 10.1371/journal.pone.0013463 20976160 PMC2956689

[B262] Sherer T. B. Betarbet R. Stout A. K. Lund S. Baptista M. Panov A. V.. (2002). An *in vitro* model of Parkinson's disease: linking mitochondrial impairment to altered alpha-synuclein metabolism and oxidative damage. J. Neurosci. 22, 7006–7015. 12177198 10.1523/JNEUROSCI.22-16-07006.2002 PMC6757862

[B263] Shirani A. Okuda D. T. Stüve O. (2016). Therapeutic advances and future prospects in progressive forms of multiple sclerosis. Neurotherapeutics 13, 58–69. 10.1007/s13311-015-0409-z 26729332 PMC4720678

[B264] Sidrauski C. Walter P. (1997). The Transmembrane kinase Ire1p is a site-specific endonuclease that initiates mRNA splicing in the unfolded protein response. Cell 90, 1031–1039. 10.1016/S0092-8674(00)80369-4 9323131

[B265] Sierra A. Gottfried-Blackmore A. C. McEwen B. S. Bulloch K. (2007). Microglia derived from aging mice exhibit an altered inflammatory profile. Glia 55, 412–424. 10.1002/glia.20468 17203473

[B266] Smith A. R. Smith R. G. Condliffe D. Hannon E. Schalkwyk L. Mill J.. (2016). Increased DNA methylation near TREM2 is consistently seen in the superior temporal gyrus in Alzheimer's disease brain. Neurobiol. Aging 47, 639.e7–639.e13. 10.1016/j.neurobiolaging.2016.07.008 27522519 PMC5747529

[B267] Smith B. N. Ticozzi N. Fallini C. Gkazi A. S. Topp S. Kenna K. P.. (2014). Exome-wide rare variant analysis identifies TUBA4A mutations associated with familial ALS. Neuron 84, 324–331. 10.1016/j.neuron.2014.09.027 25374358 PMC4521390

[B268] Smith H. L. Mallucci G. R. (2016). The unfolded protein response: mechanisms and therapy of neurodegeneration. Brain 139, 2113–2121. 10.1093/brain/aww101 27190028 PMC4958893

[B269] Smith M. A. Richey Harris P. L. Sayre L. M. Beckman J. S. Perry G. (1997). Widespread peroxynitrite-mediated damage in Alzheimer's disease. J. Neurosci. 17, 2653–2657. 9092586 10.1523/JNEUROSCI.17-08-02653.1997 PMC6573097

[B270] Snowden S. G. Ebshiana A. A. Hye A. An Y. Pletnikova O. O'Brien R.. (2017). Association between fatty acid metabolism in the brain and Alzheimer disease neuropathology and cognitive performance: a nontargeted metabolomic study. PLoS Med. 14, e1002266–e1002219. 10.1371/journal.pmed.1002266 28323825 PMC5360226

[B271] Sofic E. Lange K. W. Jellinger K. Riederer P. (1992). Reduced and oxidized glutathione in the substantia nigra of patients with Parkinson's disease. Neurosci. Lett. 142, 128–130. 10.1016/0304-3940(92)90355-B 1454205

[B272] Sorci L. Cimadamore F. Scotti S. Petrelli R. Cappellacci L. Franchetti P.. (2007). Initial-rate kinetics of human NMN-adenylyltransferases: substrate and metal ion specificity, inhibition by products and multisubstrate analogues, and isozyme contributions to NAD^+^ biosynthesis. Biochemistry 46, 4912–4922. 10.1021/bi6023379 17402747

[B273] Speakman J. R. Mitchell S. E. (2011). Caloric restriction. Mol. Aspects Med. 32, 159–221. 10.1016/j.mam.2011.07.001 21840335

[B274] Steffan J. S. Bodai L. Pallos J. Poelman M. McCampbell A. Apostol B. L.. (2001). Histone deacetylase inhibitors arrest polyglutamine-dependent neurodegeneration in Drosophila. Nature 413, 739–743. 10.1038/35099568 11607033

[B275] Stoll G. Trapp B. D. Griffin J. W. (1989). Macrophage function during Wallerian degeneration of rat optic nerve: clearance of degenerating myelin and Ia expression. J. Neurosci. 9, 2327–2335. 2787393 10.1523/JNEUROSCI.09-07-02327.1989 PMC6569766

[B276] Stranahan A. M. Mattson M. P. (2012). Recruiting adaptive cellular stress responses for successful brain ageing. Nat. Rev. Neurosci. 13, 209–216. 10.1038/nrn3151 22251954 PMC4084510

[B277] Stratmann M. Suter D. M. Molina N. Naef F. Schibler U. (2012). Circadian Dbp transcription relies on highly dynamic BMAL1-CLOCK interaction with E boxes and requires the proteasome. Mol. Cell 48, 277–287. 10.1016/j.molcel.2012.08.012 22981862

[B278] Su K. G. Savino C. Marracci G. Chaudhary P. Yu X. Morris B.. (2012). Genetic inactivation of the p66 isoform of ShcA is neuroprotective in a murine model of multiple sclerosis. Eur. J. Neurosci. 35, 562–571. 10.1111/j.1460-9568.2011.07972.x 22277070 PMC3279590

[B279] Suberbielle E. Djukic B. Evans M. Kim D. H. Taneja P. Wang X.. (2015). DNA repair factor BRCA1 depletion occurs in Alzheimer brains and impairs cognitive function in mice. Nat. Commun. 6, 8897. 10.1038/ncomms9897 26615780 PMC4674776

[B280] Sugeno N. Jackel S. Voigt A. Wassouf Z. Schulze-Hentrich J. Kahle P. J. (2016). alpha-Synuclein enhances histone H3 lysine-9 dimethylation and H3K9me2-dependent transcriptional responses. Sci. Rep. 6:36328. 10.1038/srep36328 27808254 PMC5093762

[B281] Summers D. W. Gibson D. A. DiAntonio A. Milbrandt J. (2016). SARM1-specific motifs in the TIR domain enable NAD^+^ loss and regulate injury-induced SARM1 activation. Proc. Natl. Acad. Sci. U.S.A. 113, E6271–E6280. 10.1073/pnas.1601506113 27671644 PMC5068253

[B282] Sun D. Luo M. Jeong M. Rodriguez B. Xia Z. Hannah R.. (2014). Epigenomic profiling of young and aged HSCs reveals concerted changes during aging that reinforce self-renewal. Stem Cell 14, 673–688. 10.1016/j.stem.2014.03.002 24792119 PMC4070311

[B283] Suzuki K. Koike T. (2007). Mammalian Sir2-related protein (SIRT) 2–mediated modulation of resistance to axonal degeneration in slow Wallerian degeneration mice: a crucial role of tubulin deacetylation. Neuroscience 147, 599–612. 10.1016/j.neuroscience.2007.04.059 17574768

[B284] Tagliaferro P. Burke R. E. (2016). Retrograde axonal degeneration in Parkinson Disease. JPD 6, 1–15. 10.3233/JPD-150769 27003783 PMC4927911

[B285] Tang B. L. (2016). Could sirtuin activities modify ALS onset and progression? Cell. Mol. Neurobiol. 594, 78–14. 10.1007/s10571-016-0452-2 PMC11482121 27942908

[B286] Tieu K. Perier C. Caspersen C. Teismann P. Wu D.-C. Yan S.-D.. (2003). D-β-hydroxybutyrate rescues mitochondrial respiration and mitigates features of parkinson disease. J. Clin. Invest. 112, 892–901. 10.1172/JCI200318797 12975474 PMC193668

[B287] Tofaris G. K. (2006). Pathological changes in dopaminergic nerve cells of the substantia nigra and olfactory bulb in mice transgenic for truncated human-synuclein(1-120): implications for lewy body disorders. J. Neurosci. 26, 3942–3950. 10.1523/JNEUROSCI.4965-05.2006 16611810 PMC6673887

[B288] Turkiew E. Falconer D. Reed N. Hoke A. (2017). Deletion of Sarm1 gene is neuroprotective in two models of peripheral neuropathy. J. Peripher. Nerv. Syst. [Epub ahead of print]. 10.1111/jns.12219 28485482 PMC5585053

[B289] Unger M. S. Marschallinger J. Kaindl J. Höfling C. Rossner S. Heneka M. T.. (2016). Early changes in hippocampal neurogenesis in transgenic mouse models for Alzheimer's Disease. Mol. Neurobiol. 53, 5796–5806. 10.1007/s12035-016-0018-9 27544234 PMC5012146

[B290] Valdes P. Mercado G. Vidal R. L. Molina C. Parsons G. Court F. A.. (2014). Control of dopaminergic neuron survival by the unfolded protein response transcription factor XBP1. Proc. Natl. Acad. Sci. U.S.A. 111, 6804–6809. 10.1073/pnas.1321845111 24753614 PMC4020088

[B291] Valenzuela V. Collyer E. Armentano D. Parsons G. B. Court F. A. Hetz C. (2012). Activation of the unfolded protein response enhances motor recovery after spinal cord injury. Cell Death Dis. 3, 272–279. 10.1038/cddis.2012.8 22337234 PMC3288350

[B292] Valenzuela V. Martínez G. Duran-Aniotz C. Hetz C. (2016). Gene therapy to target ER stress in brain diseases. Brain Res. 1648(Pt. B), 561–570. 10.1016/j.brainres.2016.04.064 27131987

[B293] Van der Auwera I. Wera S. Van Leuven F. Henderson S. T. (2005). Nutrition & metabolism. Nutr. Metab. 2, 28–28. 10.1186/1743-7075-2-28 PMC1282589 16229744

[B294] Vande Velde C. Garcia M. L. Yin X. Trapp B. D. Cleveland D. W. (2004). The neuroprotective factor Wlds does not attenuate mutant SOD1-mediated motor neuron disease. Neuromol. Med. 5, 193–203. 10.1385/NMM:5:3:193 15626820

[B295] Vidal R. L. Figueroa A. Court F. A. Thielen P. Molina C. Wirth C.. (2012). Targeting the UPR transcription factor XBP1 protects against Huntington's disease through the regulation of FoxO1 and autophagy. Hum. Mol. Genet. 21, 2245–2262. 10.1093/hmg/dds040 22337954 PMC3335312

[B296] Videnovic A. Lazar A. S. Barker R. A. Overeem S. (2014). “The clocks that time us”—circadian rhythms in neurodegenerative disorders. Nat. Rev. Neurol. 10, 683–693. 10.1038/nrneurol.2014.206 25385339 PMC4344830

[B297] Vilchez D. Morantte I. Liu Z. Douglas P. M. Merkwirth C. Ana P. C. Rodrigues. (2012). RPN-6 determines *C. elegans* longevity under proteotoxic stress conditions. Nature 489, 263–268. 10.1038/nature11315 22922647

[B298] Villegas R. Martinez N. W. Lillo J. Pihan P. Hernandez D. Twiss J. L.. (2014). Calcium release from intra-axonal endoplasmic reticulum leads to axon degeneration through mitochondrial dysfunction. J. Neurosci. 34, 7179–7189. 10.1523/JNEUROSCI.4784-13.2014 24849352 PMC4028495

[B299] Viswanathan M. Kim S. K. Berdichevsky A. Guarente L. (2005). A role for SIR-2.1 regulation of ER stress response genes in determining *C. elegans* life span. Dev. Cell 9, 605–615. 10.1016/j.devcel.2005.09.017 16256736

[B300] Volpicelli-Daley L. A. Luk K. C. Patel T. P. Tanik S. A. Riddle D. M. Stieber A.. (2011). Exogenous α-synuclein fibrils induce lewy body pathology leading to synaptic dysfunction and neuron death. Neuron 72, 57–71. 10.1016/j.neuron.2011.08.033 21982369 PMC3204802

[B301] Wakatsuki S. Tokunaga S. Shibata M. Araki T. (2017). GSK3B-mediated phosphorylation of MCL1 regulates axonal autophagy to promote Wallerian degeneration. J. Cell Biol. 216, 477–493. 10.1083/jcb.201606020 28053206 PMC5294778

[B302] Walther D. M. Kasturi P. Zheng M. Pinkert S. Vecchi G. Ciryam P.. (2015). Widespread proteome remodeling and aggregation in aging *C. elegans*. Cell 161, 919–932. 10.1016/j.cell.2015.03.032 25957690 PMC4643853

[B303] Wang M. C. Bohmann D. Jasper H. (2005). JNK extends life span and limits growth by antagonizing cellular and organism-wide responses to insulin signaling. Cell 121, 115–125. 10.1016/j.cell.2005.02.030 15820683

[B304] Wang T. A. Yu Y. V. Govindaiah G. Ye X. Artinian L. Coleman T. P.. (2012). Circadian rhythm of redox state regulates excitability in suprachiasmatic nucleus neurons. Science 337, 839–842. 10.1126/science.1222826 22859819 PMC3490628

[B305] Wang T. Hay J. C. (2015). Alpha-synuclein toxicity in the early secretory pathway: how it drives neurodegeneration in parkinsons disease. Front. Neurosci. 9:433. 10.3389/fnins.2015.00433 26617485 PMC4641903

[B306] Wang X. Su B. Lee H. G. Li X. Perry G. Smith M. A.. (2009). Impaired balance of mitochondrial fission and fusion in Alzheimer's Disease. J. Neurosci. 29, 9090–9103. 10.1523/JNEUROSCI.1357-09.2009 19605646 PMC2735241

[B307] Wei M. Brandhorst S. Shelehchi M. Mirzaei H. Cheng C. W. Budniak J.. (2017). Fasting-mimicking diet and markers/risk factors for aging, diabetes, cancer, and cardiovascular disease. Sci. Transl. Med. 9:eaai8700. 10.1126/scitranslmed.aai8700 28202779 PMC6816332

[B308] Weinreb R. N. Leung C. K. S. Crowston J. G. Medeiros F. A. Friedman D. S. Wiggs J. L.. (2016). Primary open-angle glaucoma. Nat. Rev. Dis. Primers 2, 16067–16019. 10.1038/nrdp.2016.67 27654570

[B309] Williamson T. L. Cleveland D. W. (1999). Slowing of axonal transport is a very early event in the toxicity of ALS-linked SOD1 mutants to motor neurons. Nat. Neurosci. 2, 50–56. 10.1038/4553 10195180

[B310] Wishart T. M. Rooney T. M. Lamont D. J. Wright A. K. Morton A. J. Jackson M.. (2012). Combining comparative proteomics and molecular genetics uncovers regulators of synaptic and axonal stability and degeneration *in vivo*. PLoS Genet. 8:e1002936. 10.1371/journal.pgen.1002936 22952455 PMC3431337

[B311] Wood H. (2013). Neurodegenerative disease: altered DNA methylation and RNA splicing could be key mechanisms in Huntington disease. Nat. Rev. Neurol. 9, 119–119. 10.1038/nrneurol.2013.23 23399643

[B312] Wormser U. Mandrioli J. Vinceti M. Fini N. Sintov A. Brodsky B. (2016). Reduced levels of alpha-1-antitrypsin in cerebrospinal fluid of amyotrophic lateral sclerosis patients: a novel approach for a potential treatment. J. Neuroinflammation 13:131 10.1186/s12974-016-0589-4 PMC4888657 27245439

[B313] Wu P. Shen Q. Dong S. Xu Z. Tsien J. Z. Hu Y. (2008). Calorie restriction ameliorates neurodegenerative phenotypes in forebrain-specific presenilin-1 and presenilin-2 double knockout mice. Neurobiol. Aging 29, 1502–1511. 10.1016/j.neurobiolaging.2007.03.028 17499883

[B314] Wu Y. Zhang S. Xu Q. Zou H. Zhou W. Cai F.. (2016). Regulation of global gene expression and cell proliferation by APP. Sci. Rep. 6:22460. 10.1038/srep22460 26936520 PMC4776145

[B315] Xi Z. Zhang M. Bruni A. C. Maletta R. G. Colao R. Fratta P.. (2015). The C9orf72 repeat expansion itself is methylated in ALS and FTLD patients. Acta Neuropathol. 129, 715–727. 10.1007/s00401-015-1401-8 25716178

[B316] Xiao A.-W. He J. Wang Q. Luo Y. Sun Y. Zhou Y.-P.. (2011). The origin and development of plaques and phosphorylated tau are associated with axonopathy in Alzheimer's disease. Neurosci. Bull. 27, 287–299. 10.1007/s12264-011-1736-7 21934724 PMC5560317

[B317] Xu L. Ryugo D. K. Pongstaporn T. Johe K. Koliatsos V. E. (2009). Human neural stem cell grafts in the spinal cord of SOD1 transgenic rats: differentiation and structural integration into the segmental motor circuitry. J. Comp. Neurol. 514, 297–309. 10.1002/cne.22022 19326469 PMC2727711

[B318] Xu L. Zhou S. Feng G.-Y. Zhang L.-P. Zhao D.-M. Sun Y.. (2012). Neural stem cells enhance nerve regeneration after sciatic nerve injury in rats. Mol. Neurobiol. 46, 265–274. 10.1007/s12035-012-8292-7 22806359

[B319] Yang L. Li S. Miao L. Huang H. Liang F. Teng X.. (2016). Rescue of glaucomatous neurodegeneration by differentially modulating neuronal endoplasmic reticulum stress molecules. J. Neurosci. 36, 5891–5903. 10.1523/JNEUROSCI.3709-15.2016 27225776 PMC4879204

[B320] Ye S. M. Johnson R. W. (1999). Increased interleukin-6 expression by microglia from brain of aged mice. J. Neuroimmunol. 93, 139–148. 10.1016/S0165-5728(98)00217-3 10378877

[B321] Yin Z. Raj D. Saiepour N. Van Dam D. Brouwer N. Holtman I. R.. (2017). Immune hyperreactivity of Aβ plaque-associated microglia in Alzheimer's disease. Neurobiol. Aging. 55, 115–122. 10.1016/j.neurobiolaging.2017.03.021 28434692

[B322] Ying Z. Zhai R. McLean N. A. Johnston J. M. Misra V. Verge V. M. K. (2015). The unfolded protein response and cholesterol biosynthesis link luman/CREB3 to regenerative axon growth in sensory neurons. J. Neurosci. 35, 14557–14570. 10.1523/JNEUROSCI.0012-15.2015 26511246 PMC6605466

[B323] Zhang B. Carroll J. Trojanowski J. Q. Yao Y. Iba M. Potuzak J. S.. (2012). The microtubule-stabilizing agent, epothilone D, reduces axonal dysfunction, neurotoxicity, cognitive deficits, and Alzheimer-like pathology in an interventional study with aged tau transgenic mice. J. Neurosci. 32, 3601–3611. 10.1523/JNEUROSCI.4922-11.2012 22423084 PMC3321513

[B324] Zhao J. Zhu Y. Yang J. Li L. Wu H. De Jager P. L.. (2017). A genome-wide profiling of brain DNA hydroxymethylation in Alzheimer's disease. Alzheimers Dement. 13, 674–688. 10.1016/j.jalz.2016.10.004 28089213 PMC6675576

[B325] Zhao W. Varghese M. Vempati P. Dzhun A. Cheng A. Wang J.. (2012). Caprylic triglyceride as a novel therapeutic approach to effectively improve the performance and attenuate the symptoms due to the Motor neuron loss in ALS disease. PLoS ONE 7:e49191. 10.1371/journal.pone.0049191 23145119 PMC3492315

[B326] Zhao Z. Lange D. J. Voustianiouk A. MacGrogan D. Ho L. Suh J.. (2006). A ketogenic diet as a potential novel therapeutic intervention in amyotrophic lateral sclerosis. BMC Neurosci. 7:29. 10.1186/1471-2202-7-29 16584562 PMC1488864

[B327] Zou Y. Jung K. J. Kim J. W. Yu B. P. Chung H. Y. (2004). Alteration of soluble adhesion molecules during aging and their modulation by calorie restriction. FASEB J. 18, 320–322. 10.1096/fj.03-0849fje 14688195

[B328] Zuleta A. Vidal R. L. Armentano D. Parsons G. Hetz C. (2012). AAV-mediated delivery of the transcription factor XBP1s into the striatum reduces mutant Huntingtin aggregation in a mouse model of Huntington's disease. Biochem. Biophys. Res. Commun. 420, 558–563. 10.1016/j.bbrc.2012.03.033 22445760

